# Dynamics of Rad9 Chromatin Binding and Checkpoint Function Are Mediated by Its Dimerization and Are Cell Cycle–Regulated by CDK1 Activity

**DOI:** 10.1371/journal.pgen.1001047

**Published:** 2010-08-05

**Authors:** Magda Granata, Federico Lazzaro, Daniele Novarina, Davide Panigada, Fabio Puddu, Carla Manuela Abreu, Ramesh Kumar, Muriel Grenon, Noel F. Lowndes, Paolo Plevani, Marco Muzi-Falconi

**Affiliations:** 1 Dipartimento di Scienze Biomolecolari e Biotecnologie, Università degli Studi di Milano, Milano, Italy; 2 Centre for Chromosome Biology, School of Natural Science, National University of Ireland Galway, Galway, Ireland; The University of North Carolina at Chapel Hill, United States of America

## Abstract

*Saccharomyces cerevisiae* Rad9 is required for an effective DNA damage response throughout the cell cycle. Assembly of Rad9 on chromatin after DNA damage is promoted by histone modifications that create docking sites for Rad9 recruitment, allowing checkpoint activation. Rad53 phosphorylation is also dependent upon BRCT-directed Rad9 oligomerization; however, the crosstalk between these molecular determinants and their functional significance are poorly understood. Here we report that, in the G1 and M phases of the cell cycle, both constitutive and DNA damage-dependent Rad9 chromatin association require its BRCT domains. In G1 cells, GST or FKBP dimerization motifs can substitute to the BRCT domains for Rad9 chromatin binding and checkpoint function. Conversely, forced Rad9 dimerization in M phase fails to promote its recruitment onto DNA, although it supports Rad9 checkpoint function. In fact, a parallel pathway, independent on histone modifications and governed by CDK1 activity, allows checkpoint activation in the absence of Rad9 chromatin binding. CDK1-dependent phosphorylation of Rad9 on Ser11 leads to specific interaction with Dpb11, allowing Rad53 activation and bypassing the requirement for the histone branch.

## Author Summary

In response to DNA damage all eukaryotic cells activate a surveillance mechanism, known as the DNA damage checkpoint, which delays cell cycle progression and modulates DNA repair. Yeast *RAD9* was the first DNA damage checkpoint gene identified. The genetic tools available in this model system allow to address relevant questions to understand the molecular mechanisms underlying the Rad9 biological function. By chromatin-binding and domain-swapping experiments, we found that Rad9 is recruited into DNA both in unperturbed and in DNA–damaging conditions, and we identified the molecular determinants required for such interaction. Moreover, the extent of chromatin-bound Rad9 is regulated during the cell cycle and influences its role in checkpoint activation. In fact, the checkpoint function of Rad9 in G1 cells is solely mediated by its interaction with modified histones, while in M phase it occurs through an additional scaffold protein, named Dpb11. Productive Rad9-Dpb11 interaction in M phase requires Rad9 phosphorylation by CDK1, and we identified the Ser11 residue as the major CDK1 target. The model of Rad9 action that we are presenting can be extended to other eukaryotic organisms, since Rad9 and Dpb11 have been conserved through evolution from yeast to mammalian cells.

## Introduction

The DNA damage checkpoint coordinates cell cycle progression, DNA repair, replication, recombination, apoptosis and senescence in response to genotoxic stress. Defects in this surveillance mechanism lead to increased genomic instability, cancer susceptibility, ageing and several human pathologies [1], [2]. The checkpoint is organized as a signal transduction cascade, whose players have been conserved throughout evolution [3], [4]. When DNA is damaged, cells are able to sense and process the lesions generating a series of phosphorylation events, which are then amplified and propagated to specific targets [3], [4]. Critical checkpoint factors are phosphorylated in response to DNA damage and their order of functions in the cascade has been mainly inferred by monitoring their phosphorylation state [5]. The apical kinases in the pathway are members of a family of phosphatidylinositol 3′ kinase-like kinases (PIKKs), which includes Mec1 and Tel1 from budding yeast, as well as mammalian ATM, ATR and DNA-PK [6]. In the yeast *Saccharomyces cerevisiae* the first biochemical event in response to checkpoint activation is the Mec1-dependent phosphorylation of its interacting subunit Ddc2 [7]–[9]. Other critical Mec1 targets are histone H2A, the 9-1-1 complex and the Rad9 mediator which is necessary for the recruitment and activation of the main effector kinase Rad53 [10]–[16]. Rad53 phosphorylation is a key step in the signal transduction cascade and it is generally used as a marker to monitor full checkpoint activation [17].

In a pioneering study, *RAD9* was the first DNA damage checkpoint gene identified in yeast and it is required for proper DNA damage response in all cell cycle phases and in response to a variety of genotoxins [18]–[20]. Rad9 is a large protein of 148 kDa containing a tandem repeat of the BRCT (BRCA1 C-terminus) motif, which is required for Rad9 oligomerization and function [21]–[23]. Until recently the biochemical role of the *RAD9* gene product remained obscure. Gilbert et al., were the first to purify Rad9 complexes from undamaged and UV-treated cells; structural characterization of such complexes led to the proposal that Rad9 recruits and catalyzes the activation of Rad53, by acting as a scaffold protein bringing Rad53 molecules in close proximity, thus facilitating the Rad53 autophosphorylation reaction [14].

The Rad9 protein contains several potential target sites for CDK1/Cdc28 kinase and PIKK-directed phosphorylation [24]. Rad9 is phosphorylated in an unperturbed cell cycle and it is hyper-phosphorylated in a Mec1- and/or Tel1-dependent manner after genotoxic treatments [12], [13]. This hyper-phosphorylation is a pre-requisite for Rad9-Rad53 association, which is mediated by the two forkhead associated (FHA) Rad53 domains and specific Rad9 amino acid residues that are modified in the hyper-phosphorylated Rad9 form [12], [13], [15], [16], [25]–[27]. Recent data confirmed that the Rad9 BRCT domains mediate Rad9 oligomerization, and these interactions are also modulated by Mec1/Tel1-dependent phosphorylation of a SQ/TQ cluster domain (SCD) in Rad9. Rad9 oligomerization is required to maintain checkpoint signaling through a feedback loop involving Rad53-dependent phosphorylation of the Rad9 BRCT domains, which attenuates BRCT-SCD interactions [27].

Despite the fundamental nature of the cellular response to DNA damage, Rad9 and its *Schizosaccharomyces pombe* and metazoan orthologs Crb2 and 53BP1 show a modest level of amino acid sequence conservation. Dimerization mediated by the BRCT domains has been shown to be essential for the biological function of both Rad9 and Crb2 [21], [28], however, 53BP1 oligomerization occurs in a BRCT-independent manner [29], [30]. Recent structural analysis showed that an equivalent surface is conserved to a certain degree also in 53BP1 and it provides the binding site for p53. It was thus suggested that a functional requirement for dimerization of a checkpoint mediator may have been conserved in the evolution, but in metazoan organisms it may be delivered via a second protein rather than through homotypic interactions [31].

In the last few years it became evident that chromatin remodelling activities and post-translational modifications of chromatin components, including histones, influence DNA damage checkpoint signalling and repair in all eukaryotic cells (see [32] for a recent review). Moreover, it has been recently suggested that Rad9 may also be chromatin-bound in the absence of DNA damage [22]. This dynamic interaction with chromatin appears to require the Tudor domain of Rad9 and methylated lysine 79 of histone H3 (H3-K79me). Furthermore, this interaction modulates Rad9 functions after DNA damage [22], [23], [33]–[35]. However, the Crb2 and 53BP1 orthologues of Rad9 both recognize H4 methylated at lysine 20 (H4-K20me), although human 53BP1 may also be recruited to chromatin through interactions with H3-K79me [34], [36]–[39].

For the Rad9/Crb2/53BP1 mediator proteins, efficient recruitment seems to require additional molecular interactions. Rad9 and Crb2 interact via their BRCT domains with H2A phosphorylated at serine 129 (γH2A) at sites of DNA damage [22], [31], [37], [40]–[42]. 53BP1 binding to DSBs is facilitated by phosphorylation of serine 139 of the histone variant H2AX (γH2AX) [29], [43]–[45]. It has been reported that various oligomerization domains in 53BP1 facilitate its recruitment to damaged DNA sites [30]. Moreover, 53BP1 recruitment to chromatin is facilitated by ubiquitination of H2A and H2AX by RNF8 through a yet unidentified mechanism [46]–[48].

Recently, it has been shown that Dpb11 in *S. cerevisiae* and its *S. pombe* and metazoan orthologs, termed Rad4/Cut5 and TopBP1, respectively, are required for full PIKK-dependent checkpoint activation in response to DNA damage [49], [50]. Moreover it has been suggested that Dpb11 orthologs may modulate checkpoint activation through interaction with mediator/adaptor proteins [37], [51]. To explore the functional role and the relationship between the BRCT domains and Rad9 ability to bind chromatin, we have analyzed both Rad9 chromatin recruitment and checkpoint activation in cells engineered to express various forms of Rad9 harboring mutated BRCT domains, including point mutations, deletion and substitutions with heterologous dimerization domains. We found that the requirements for Rad9 binding to chromatin are different in G1 or in M phase cells and in damaging versus unperturbed conditions. Moreover, we tested the requirements for Rad9 chromatin binding in yeast mutants defective in either the histone-dependent and/or histone-independent pathways essential for full checkpoint activation in M phase. Importantly, we found that CDK1-dependent Rad9 phosphorylation on Ser11 modulates the Dpb11-dependent branch in the M phase of the cell cycle in a chromatin-independent manner.

## Results

### Rad9 BRCT domains are required for its binding to chromatin in unperturbed and DNA damaging conditions

The Rad9 checkpoint mediator protein contains a tandem repeat of the BRCT motif at its C-terminus. Previous experiments have shown that the BRCT domains are critical for the activation of the DNA damage checkpoint and two-hybrid and GST pull-down analysis indicated that the BRCT domains modulate Rad9-Rad9 interactions [21]. More recently, it has been shown that Rad9 mutations in a conserved region of the first BRCT motif affect binding to γH2A, thus altering the G1 checkpoint signaling in response to DSBs [22], [40] and the G2/M response to uncapped telomeres [23]. However, the mutations analyzed did not influence Rad9 chromatin binding in unperturbed conditions [22].

**Figure 1:**
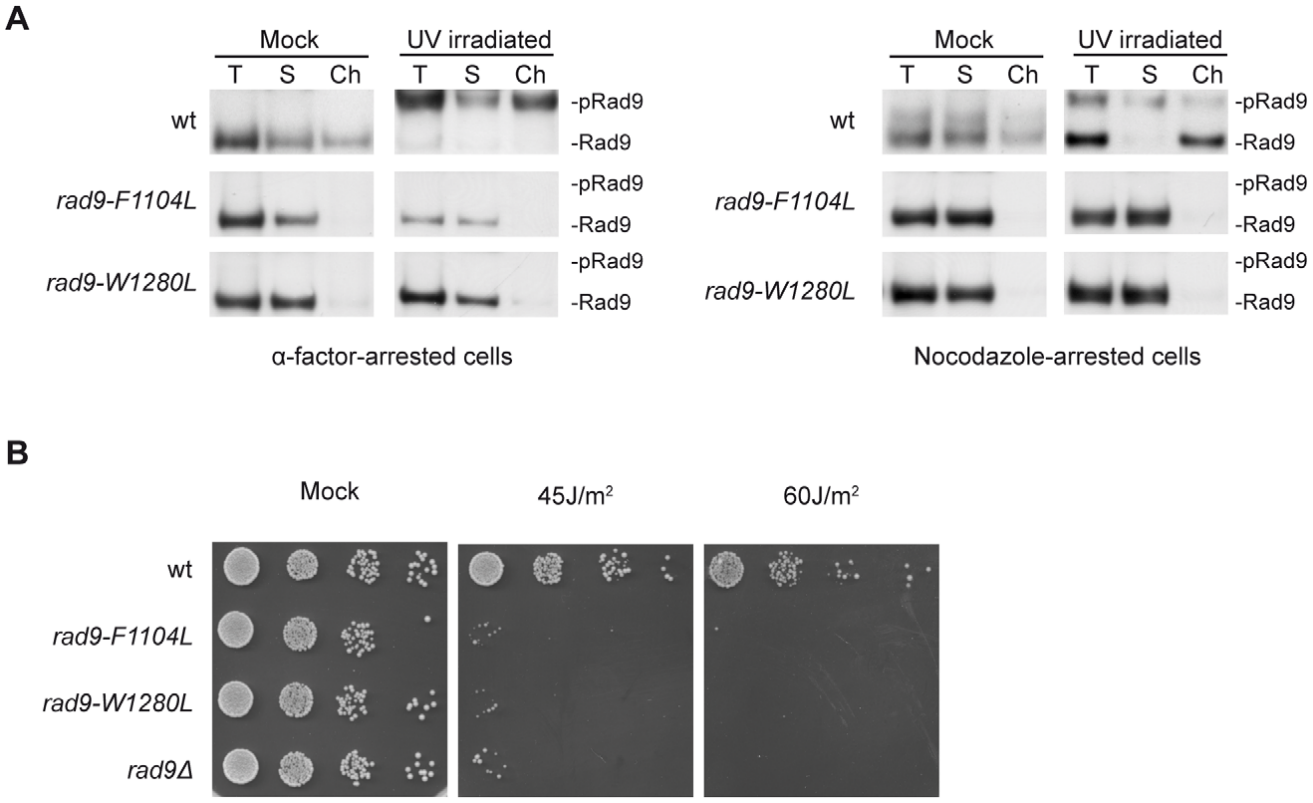
Rad9 chromatin binding requires an intact BRCT domains in UV–treated and in unperturbed conditions. (A) wt (K699), *rad9-F1104L* (YNOV15), *rad9-W1280L* (YNOV31) strains were arrested in G1 with α-factor or in M with nocodazole and either mock or UV irradiated (75 J/m^2^). 10 min after irradiation, samples were collected and analyzed in their total (T), soluble (S) and chromatin-enriched (Ch) fractions. Blots were probed with anti-Rad9 antibodies and, after staining, the blots were cut to eliminate the Rad9-unrelated protein species migrating adjacent to the hyper-phosphorylated Rad9 isoform (Figure S1A). The positions of Rad9 and its hyper-phosphorylated isoform (pRad9) are indicated. (B) The same yeast strains analyzed in A and a *rad9Δ* strain (YMAG88) were grown overnight to log phase and serial dilutions were spotted onto YPD plates, which were then irradiated at the indicated UV doses and incubated for 3 days.

The *rad9-F1104L* or the *rad9-W1280L* mutations substitute the most highly conserved amino acid residues in the two BRCT motifs and each mutation affects productive Rad9-Rad9 interactions [21]. We tested whether such *rad9* mutations impair Rad9 recruitment to chromatin both in unperturbed and DNA damaging conditions. As expected, a proportion of wild-type Rad9 migrated much more slowly under our gel running conditions after UV treatment, consistent with hyper-phosphorylation of Rad9 (Figure 1A). A relevant fraction of Rad9 was found associated to chromatin in the absence of DNA damage, both in G1- and in M-arrested cells, confirming previous observations [22]. Control experiments were routinely performed to verify the distribution of standard protein markers in the soluble and chromatin fractions (Figure S1B). In various experiments we consistently found that the ratio of hyper- to hypo-phosphorylated Rad9 was approximately constant in both the soluble and chromatin fractions in G1 cells. Interestingly, in M phase cells, hyper-phosphorylated Rad9 was mostly present in the soluble fraction, while chromatin was enriched in the hypo-phosphorylated form (Western blot quantitation are shown in Figure S1C). As shown in Figure 1A, any of the two BRCT mutations abolished Rad9 phosphorylation and recruitment to chromatin in G1- or M-arrested cells. As expected [21], *rad9-F1104L* and *rad9-W1280L* mutant cells were highly sensitive to UV treatments (Figure 1B).

These results indicate that BRCT domains influence not only Rad9 binding to chromatin by modulating its interaction with γH2A after DNA damage [22], but they also control Rad9 recruitment to chromatin in unperturbed conditions.

### A heterologous dimerization domain restores Rad9 binding to chromatin in G1-arrested, but not M-arrested, cells

To further evaluate the relevance of Rad9-Rad9 interactions in chromatin binding, we generated a set of yeast strains in which the C-terminal region of Rad9, containing the BRCT motifs, was substituted with either a 13-MYC epitope or a GST tag (see Materials and Methods). The latter has been shown to act as a heterologous constitutive dimerization domain [28], [52], [53].

**Figure 2:**
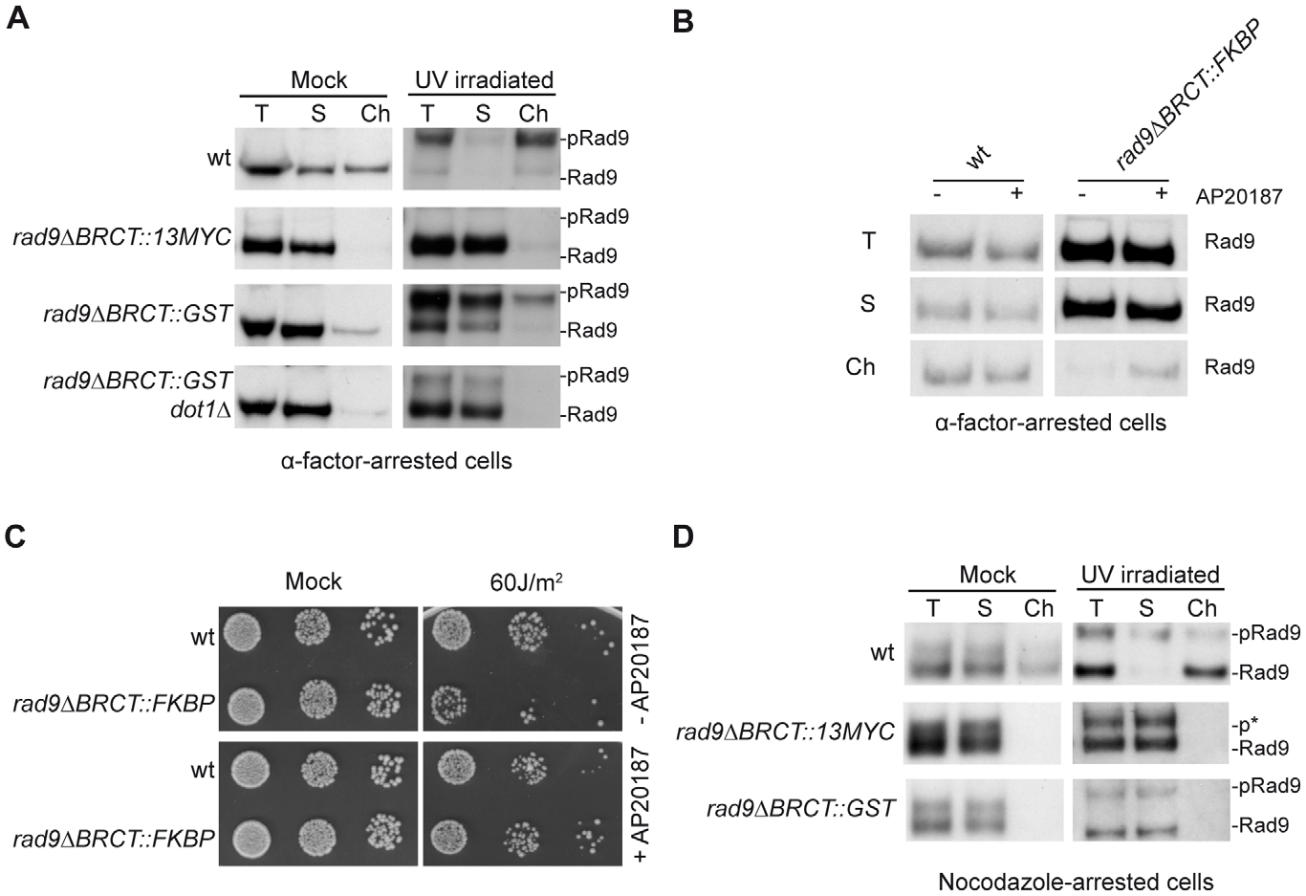
GST-driven Rad9 dimerization recovers its binding to chromatin in G1, but not in M phase. (A) wt (K699), *rad9ΔBRCT::13MYC* (YFL696/1b), *rad9ΔBRCT::GST* (YMAG74) and *rad9ΔBRCT::GST dot1Δ* (YFL773/2c) cells were arrested in G1 with α-factor and either mock or UV irradiated (75 J/m^2^). After 10 min, samples were collected and analyzed in their total (T), soluble (S) and chromatin-enriched (Ch) fractions. Blots were probed with anti Rad9 antibodies as in the legend to Figure 1A. (B) wt (K699) and *rad9ΔBRCT::FKBP* (YFL901) cells were incubated for 6 h in the presence or in the absence of the dimerization-inducing molecule AP20187, blocked in G1 with α-factor and analyzed in their total (T), soluble (S) and chromatin-enriched (Ch) fractions. Blots were probed with anti Rad9 antibodies. (C) The same strains as in B were grown overnight to log phase and incubated for 6 h in the presence or in the absence of the dimerization-inducing molecule AP20187. Serial dilutions were spotted onto YPD plates, which were then irradiated at the indicated UV doses and incubated for 3 days. (D) Western blot analysis of the total, soluble and chromatin-enriched fractions from wt (K699), *rad9ΔBRCT::13MYC* (YFL696/1b) and *rad9ΔBRCT::GST* (YMAG74) cells arrested in M phase and either mock or UV irradiated (75 J/m^2^). In all panels, the positions of Rad9 and its hyper-phosphorylated isoform (pRad9) are indicated. p* marks a partially phosphorylated Rad9 species.

As shown in Figure 2A, the GST tag was capable of driving, albeit somewhat less efficiently, Rad9 chromatin binding in G1-arrested cells, both in the absence or presence of DNA damage. Importantly, Rad9ΔBRCT::GST recruitment to chromatin still occurs through its interaction with H3-K79me, as it was drastically reduced in a *dot1Δ* background, lacking the specific H3-K79 histone methyl-transferase. Rad9 dimerization through the GST tag also significantly recovered Rad9 hyper-phosphorylation after UV irradiation and full checkpoint function (Figure 2A and data not shown).

It must be underlined that addition of the GST tag to Rad9ΔBRCT, allowing Rad9 dimerization, reconstitutes chromatin binding even though Rad9ΔBRCT::GST lacks the BRCT tandem repeats and is, therefore, unable to interact with γH2A [22]. These authors suggested that, after DNA damage, Rad9 shifts from H3-K79me to phosphorylated H2A-S129, and this translocation would be deficient in *rad9ΔBRCT::GST* cells. As a consequence of its defective interaction with γH2A, binding of Rad9ΔBRCT::GST to chromatin is probably much less stable. This hypothesis may explain the finding that in the *rad9ΔBRCT::GST* strain the majority of phosphorylated Rad9 after UV irradiation in G1 is found in the soluble fraction (Figure 2A).

To further support the role of Rad9 dimerization in its chromatin binding in G1-arrested cells solely by inducing Rad9-Rad9 interactions, we tested the possibility to direct a Rad9ΔBRCT isoform to chromatin by adding to the truncated protein a FKBP tag, which can dimerize only in the presence of the small inducing molecule AP20187 [54]. Indeed, the presence of the FKBP tag partially rescued Rad9 chromatin binding in G1-arrested cells, but only in the presence of inducing AP20187 (Figure 2B). Importantly, addition of the dimerization inducing molecule fully recovered the UV sensitivity of *rad9DBRCT* cells (Figure 2C).

**Figure 3:**
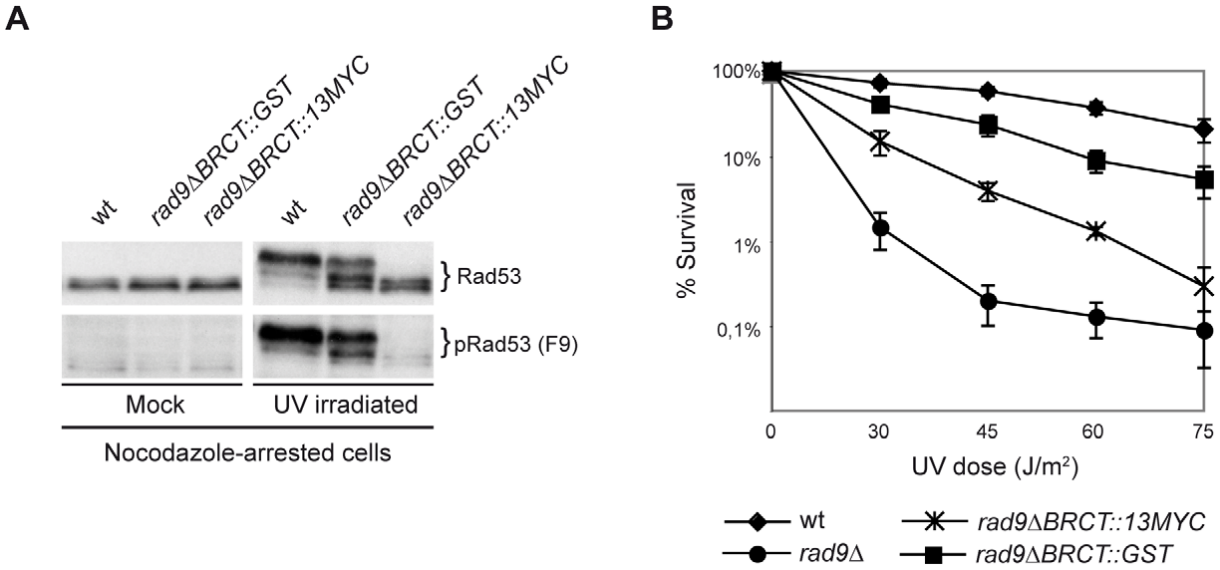
GST-driven Rad9 dimerization allows M checkpoint function regardless of Rad9 chromatin binding. (A) wt (K699), *rad9ΔBRCT::13MYC* (YFL696/1b), *rad9ΔBRCT::GST* (YMAG74) cells were cultured to mid-log phase, arrested in M with nocodazole, and either mock or UV irradiated (75 J/m^2^); 10 min after irradiation, Rad53 phosphorylation was analyzed by SDS-PAGE and Western blotting with polyclonal Rad53 antibodies and with the F9 monoclonal antibody (Mab) recognizing only the hyper-phosphorylated active form of Rad53 to monitor checkpoint activation. (B) The same cells analyzed in A and a *rad9Δ* control strain (YMAG88) were cultured overnight, diluted and plated on YPD plates, which were irradiated with the indicated UV doses. Cell survival was assayed by determining the number of colonies grown on plates after 2 days; error bars were obtained from 3 independent experiments.

Contrary to our observations in G1-arrested cells, the heterologous GST dimerization domain did not rescue Rad9 binding to chromatin in nocodazole-arrested cells, although it restored checkpoint activation after DNA damage (Figure 2D, Figure 3A). Rad9 missing the BRCT domains only exhibits partial phosphorylation; this form can be distinguished from the hyper-phosphorylated isoform due to different electrophoretic mobility and its incapacity to activate Rad53 (see Figure 3A).

Altogether, the findings reported above indicate that dimerization is required for Rad9 to bind H3-K79me in G1-arrested cells, both with and without an exogenous DNA damaging agent. However, this is not the case in M phase-arrested cells, where GST-directed Rad9 dimerization partially recovers genotoxin-induced Rad9 hyper-phosphorylation, but fails to rescue its binding to chromatin. This may suggest that, at least in M phase, Rad9 chromatin binding is not directly linked to Rad9 hyper-phosphorylation.

### GST-driven Rad9 dimerization rescues checkpoint activation and UV–sensitivity, despite undetectable chromatin binding

Although the addition of a heterologous dimerization domain to truncated Rad9ΔBRCT was not able to allow Rad9 chromatin binding in M phase-arrested cells, it rescues Rad53 activation after UV irradiation. In fact, as shown in Figure 3A, the phosphorylation state of the effector checkpoint kinase, Rad53, was found to be very different after UV-irradiation of *rad9ΔBRCT::GST* or *rad9ΔBRCT::13MYC* cells arrested with nocodazole. The hyper-phosphorylated form of Rad53 is absent in UV treated *rad9ΔBRCT::13MYC* cells, while it is clearly detectable in *rad9ΔBRCT::GST* cells. Although the extent of Rad53 phosphorylation was reduced in *rad9ΔBRCT::GST* relative to wild-type cells, the presence of the heterologous GST dimerization domain recovers the Rad9 checkpoint function, as confirmed by a direct checkpoint assay (data not shown). This conclusion is also supported by the observation that addition of the GST tag significantly rescued, although not completely, the UV sensitivity of the *rad9ΔBRCT::13MYC* strain (Figure 3B), and these findings are in agreement with previous experiments in *S.pombe* [28].

Thus far our data indicate that dimerization of Rad9 directed by an heterologous domain confers activation of the DNA damage checkpoint cascade, as well as significant resistance to UV in M phase-arrested cells, despite undetectable binding of Rad9 to chromatin (see Figure 2D).

### Checkpoint activation in M phase requires CDK1 activity and is driven by Rad9–Dpb11 interaction

**Figure 4:**
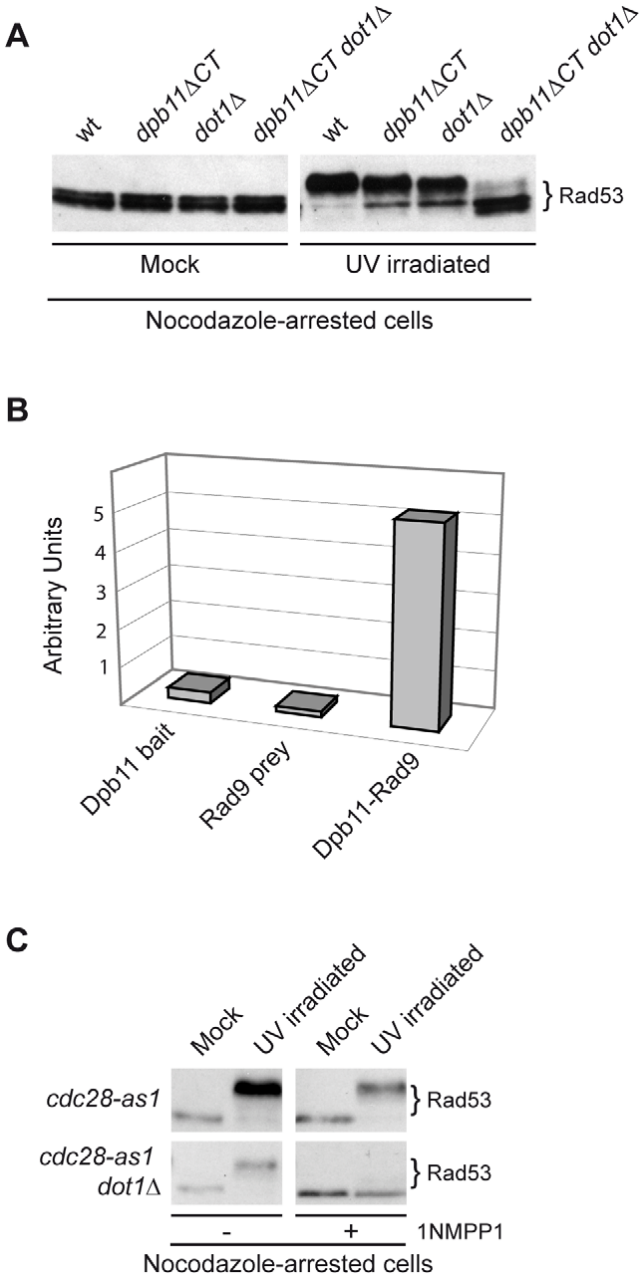
A cell cycle–dependent interaction between Dpb11 and Rad9 may regulate the Dpb11-dependent pathway. (A) wt (YMAG149/7B), *dpb11ΔCT* (YMAG145/20C), *dot1Δ* (YMAG150/4A) and *dpb11ΔCT dot1Δ* (YMAG148) strains were arrested in M with nocodazole and mock or UV irradiated (75 J/m^2^). 10 min after irradiation, samples were taken and protein extracts were separated by SDS-PAGE. Blots were analyzed with anti Rad53 antibodies. (B) EGY42 cells, containing the pSH18-34 β-galactosidase reporter plasmid, were transformed with the Rad9 prey plasmid pMAG11.1 (pJG4-5-*RAD9*) and/or with the Dpb11 bait plasmid pFP15 (pEG202-*DPB11*). Strains were cultured overnight in -His, -Trp, -Ura medium plus raffinose and arrested in M phase by nocodazole treatment. Galactose was then added to the medium to induce bait expression. A modified version of ONPG yeast two-hybrid assay was used to determine the β-galactosidase activity in each strain, expressed in relative units. (C) *cdc28-as1* (JAU01) and *cdc28-as1 dot1Δ* (YNOV4) strains were arrested in M with nocodazole and, after incubation for 2 h in the absence or in the presence of 5 µM 1NMPP1, were either mock or UV irradiated (75 J/m^2^). After 10 min, samples were collected and protein extracts were separated by SDS-PAGE. Blots were analyzed with anti-Rad53 antibodies.

We have recently demonstrated that in the M phase of the cell cycle, full activation of the DNA damage checkpoint in response to various genotoxic stress is dependent upon Dpb11 [50]. Our data suggested that Dpb11 facilitates the recruitment of Rad9 proximally to DNA lesions through a mechanism independent of histone modifications. Indeed, as shown in Figure 4A, checkpoint activation after UV irradiation of nocodazole-arrested cells is only partially affected either in *dot1Δ* or in *dpb11ΔCT* cells. On the other hand, *dot1Δ dpb11ΔCT* double mutant cells are dramatically deficient in Rad53 phosphorylation since both the histone-dependent and histone-independent pathways for checkpoint activation are not functional. This finding can be interpreted by hypothesizing that when Rad9 cannot bind to chromatin via histone marks, Dpb11 may act as a platform for Rad9 recruitment in a histone-independent manner. Moreover, because the Dpb11-dependent pathway is particularly relevant in the G2 to M phases of the cell cycle [50], it was tempting to hypothesize that the proposed interaction between Rad9 and Dpb11 might be regulated by cell cycle-dependent control mechanisms [55]. Initially, we monitored this interaction using two-hybrid analysis performed at different cell cycle stages (see Materials and Methods). As shown in Figure 4B, a strong Rad9-Dpb11 interaction was observed in nocodazole-arrested cells. Several independent two-hybrid experiments showed that Rad9-Dpb11 interaction was more evident in M- rather than in G1-arrested cells. Experiments performed with a bait and a prey already known to interact by two-hybrid, indicate that the M/G1 ratio of Rad9-Dpb11 interaction was significantly higher than that found in the controls, suggesting a cell cycle-specific effect (Figure S2A). The Rad9-Dpb11 interaction was further confirmed biochemically (see below).

Since the interaction between Rad9 and Dpb11 appears to be induced in M phase, we reasoned that the Dpb11-dependent branch of the DNA damage checkpoint in M phase might be related to the increasing level of CDK1 kinase activity as cells move through the S, G2 and M phases of the cell cycle. To address this issue, we took advantage of the *cdc28-as1* mutant (in which only the Cdc28 kinase is specifically sensitive to bulky ATP analogues, such as 1NMPP1 [56]) to conditionally inactivate CDK1 in nocodazole-treated cells. Cdc28 kinase activity was inhibited or not with 1NMPP1 in nocodazole arrested cells and mitotic cells were then mock- or UV irradiated to induce DNA damage. Western blot analysis of Rad53 revealed that CDK1 inhibition abolished phosphorylation of Rad53 in the absence of the histone-dependent pathway, while no effect was observed in *DOT1* cells (Figure 4C). A similar experiment was performed by tethering checkpoint factors to DNA in the absence of damage [57]. The difference between our result and that reported by Bonilla, may be explained if, in their experimental conditions, without the addition of genotoxic agents, checkpoint activation is independent upon the Dpb11 branch.

Altogether, our results indicate that CDK1 activity is required for the function of the histone-independent branch necessary for Rad53 phosphorylation in cells arrested in mitosis.

### CDK1-dependent phosphorylation of serine 11 of Rad9 modulates the Dpb11-dependent branch in M phase cells

**Figure 5:**
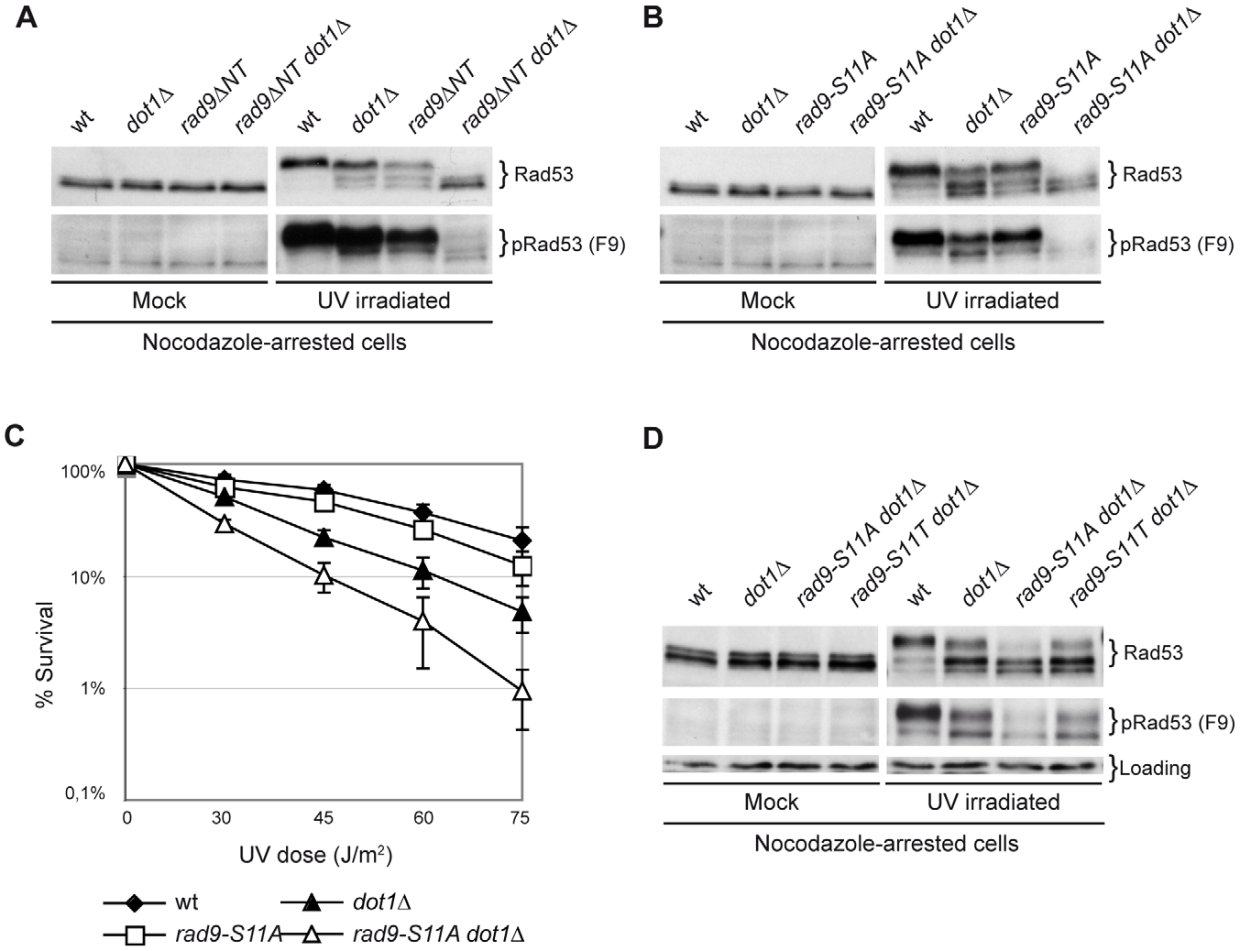
Phosphorylation of Rad9S11 by CDK1 is required for the establishment of an effective UV response in the absence of Dot1. (A) wt (K699), *dot1Δ* (YFL234), *rad9ΔNT* (DLY2236) and *rad9ΔNT dot1Δ* (YFP91) strains were arrested with nocodazole and either mock or UV irradiated (75 J/m^2^). After 10 min samples were collected and protein extracts were separated by SDS-PAGE. Blots were analyzed with anti-Rad53 or with the F9 Mab to monitor checkpoint activation. (B) wt (K699), *dot1Δ* (YFL234), *rad9-S11A* (YMAG162) and *rad9-S11A dot1Δ* (YMAG164) strains were arrested in M, irradiated and Rad53 was detected by Western blotting as describe in panel A. (C) The same strains analyzed in B were cultured overnight, diluted and plated on YPD plates, which were irradiated with the indicated UV doses. Cell survival was assayed as described in the legend of Figure 3. (D) wt (K699), *dot1Δ* (YFL234), *rad9-S11A dot1Δ* (YMAG164) and *rad9-S11T dot1Δ* (YNOV52) strains were arrested with nocodazole and either mock or UV irradiated (75 J/m^2^). After 10 min samples were collected and protein extracts were separated by SDS-PAGE. Blots were analyzed with anti-Rad53 or with the F9 Mab to monitor checkpoint activation.

Rad9 contains 20 potential (SP or TP) target sites for CDK-dependent phosphorylation, 9 of which conform to the canonical CDK phosphorylation site (S/T-P-x-K/R) (Figure S2B). We hypothesized that Rad9 could be a relevant CDK1 target in the histone-independent branch of the DNA damage checkpoint in M phase cells. Initially, we tested a *rad9ΔNT* mutant strain, in which the first 231 amino acids, including 9 S/T-P sites, of Rad9 are missing (Materials and Methods and [58]). As shown in Figure 5A, Rad53 phosphorylation was partially defective in both *dot1D* and *rad9DNT* mutants and essentially abolished in a *rad9ΔNT dot1Δ* double mutant strain.

All 9 potential Cdc28 phosphorylation sites in the Rad9 N-terminal region were individually mutagenized and different mutant combinations tested (Materials and Methods and data not shown). *rad9-S11A* cells displayed a detectable defect in cell cycle-regulated Rad9 phosphorylation (Figure S2C). Moreover, the *rad9-S11A* mutation recapitulates the phenotype we observed in *rad9ΔNT* cells, namely, severe loss of DNA damage-dependent Rad53 phosphorylation when combined with *dot1D* (Figure 5B). Consistently, the *rad9-S11A* mutation alone did not confer a strong sensitivity to UV irradiation (Figure 5C), while a *rad9-S11A dot1Δ* double mutant strain was synthetically sensitive to genotoxic treatment. On the other hand, a *rad9-S11A dpb11ΔCT* double mutant strain did not exhibit an increased sensitivity to UV irradiation when compared to strains harboring the single mutations, indicating that Dpb11 and Rad9-S11 phosphorylation act in the same pathway (data not shown). Phosphorylation of Rad9S11 has been reported *in vivo* [59]. In order to verify the relevance of S11 phosphorylation in our experimental conditions, we reverted the S11A mutation to Thr, another phosphorylatable residue. Figure 5D shows that Rad9 carrying a Thr at position 11 rescues the phenotype imparted by the S11A mutation, since checkpoint activation in the *rad9-S11T dot1Δ* strain is identical to that found in *dot1Δ* cells.

**Figure 6:**
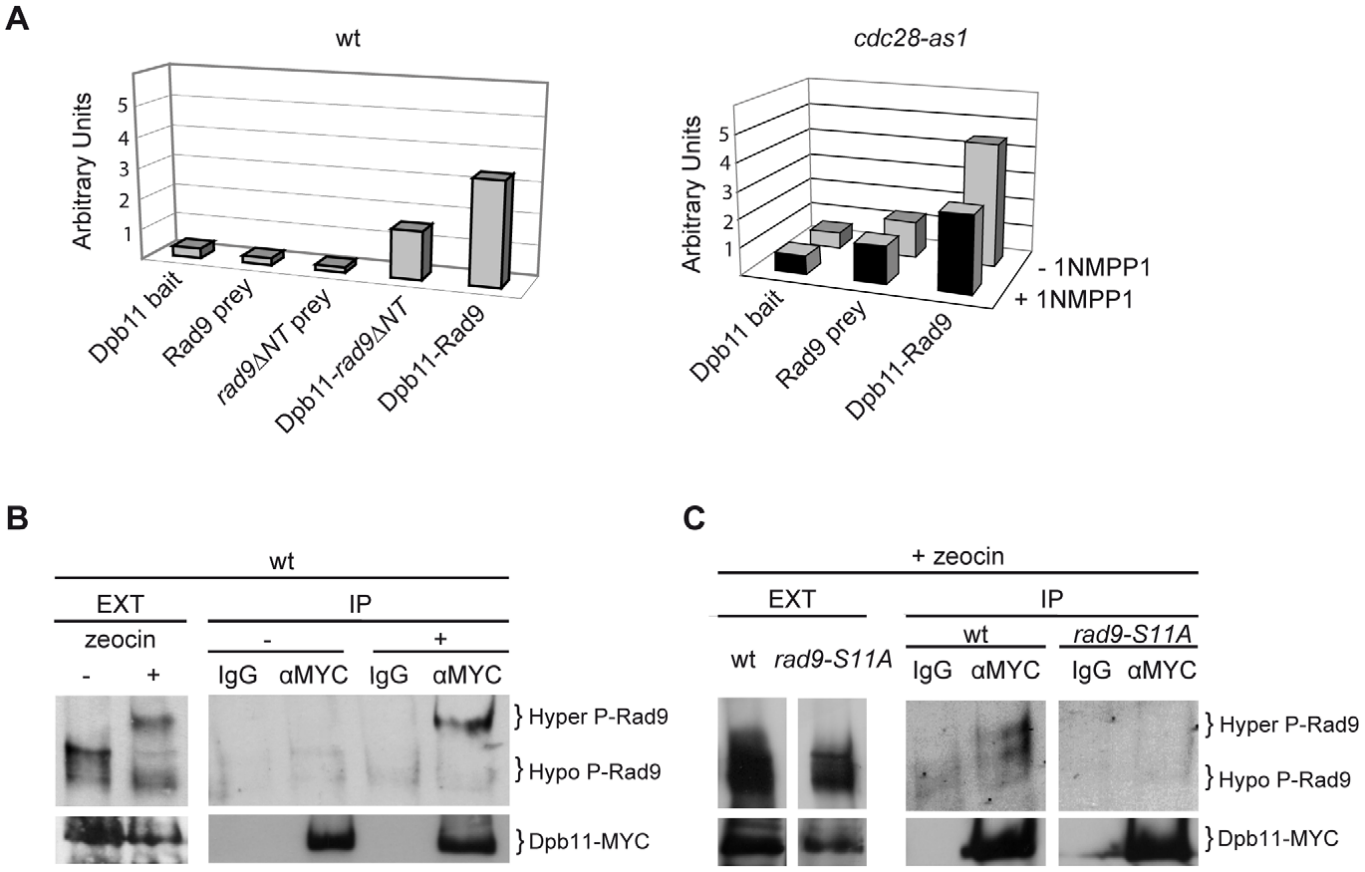
CDK1-dependent phosphorylation of S11-Rad9 modulates Rad9-Dpb11 interaction. (A) Two-hybrid interaction between Dpb11 and Rad9 was tested in a wt (K699) (left) or in a *cdc28-as1* (JAU01) (right) genetic background with the indicated bait and prey plasmids. Where specified 5 µM 1NMPP1 was added to the media for 1 h before bait induction and extracts preparation. (B) The *Dpb11-myc* (YFP38) strain was arrested with nocodazole and either mock treated or treated with 150 µg/ml of zeocin for 30 min. Whole cell protein extract was prepared and tagged Dpb11-MYC was immunoprecipitated either with anti-MYC antibodies or unspecific mouse IgG as described in Materials and Methods. The presence of Rad9 in the IPs was detected by Western blot analysis of the immunoprecipitates with specific anti-Rad9 antibodies. (C) Immunoprecipitations with anti-MYC antibodies were performed on extracts from nocodazole arrested cells, treated with 150 µg/ml of zeocin for 30 min, expressing Dpb11-MYC in a *RAD9* (YFP38) or *rad9S11A* (YMAG281) background. The presence of Rad9 was detected by Western blot analysis of the immunoprecipitates with specific anti-Rad9 antibodies. Lower exposure of the crude extracts lanes are shown to allow visualization of both Rad9 and Dpb11 specific bands.

Interestingly, Rad9-Dpb11 interaction by two-hybrid analysis was reduced when the Rad9NT isoform, lacking the 9 potential CDK1 phosphorylation sites, was used as a prey in a wild-type background, or when Cdc28 activity was inhibited by 1NMPP1 addition in the *cdc28-as1* strain (Figure 6A). The *in vivo* interaction between Rad9 and Dpb11 was also confirmed by co-immuprecipitation of the endogenous proteins after genotoxic treatment. As shown in Figure 6B, immunoprecipitation of MYC-tagged Dpb11 recovers the hyper-phosphorylated isoform of Rad9, and this interaction is virtually lost in the *rad9-S11A* mutant strain (Figure 6C). We also noticed that the Rad9-S11A mutant protein has slighlty less gel-mobility than its wild type counterpart, as shown in Figure 6C. This observation can be explained by either a mild defect in Mec1/Tel1-dependent hyperphosphorylation of the Rad9-S11A protein, due to the loss of Rad9-Dpb11 interaction, or a direct effect of the S11A mutation which, affecting CDK1-dependent phosphorylation of Rad9, may directly modify its migration in SDS PAGE.

Altogether, the above findings indicate that the Ser11 CDK1-consensus site on Rad9 is a relevant target to modulate Rad9-Dpb11 interaction and the CDK1-dependent checkpoint response in M phase cells.

### The Dpb11-dependent branch in M phase modulates checkpoint activation in a chromatin-independent manner

To gain further insights into the mechanisms involving Rad9 and the Dpb11-dependent branch of the DNA damage checkpoint operating in nocodazole-arrested cultures, cell extracts were fractionated into soluble and chromatin fractions. Specifically, we monitored Rad9 chromatin binding and Rad53 phosphorylation in strains harbouring defects in the different branches known to regulate Rad9 checkpoint functions during M phase.

**Figure 7:**
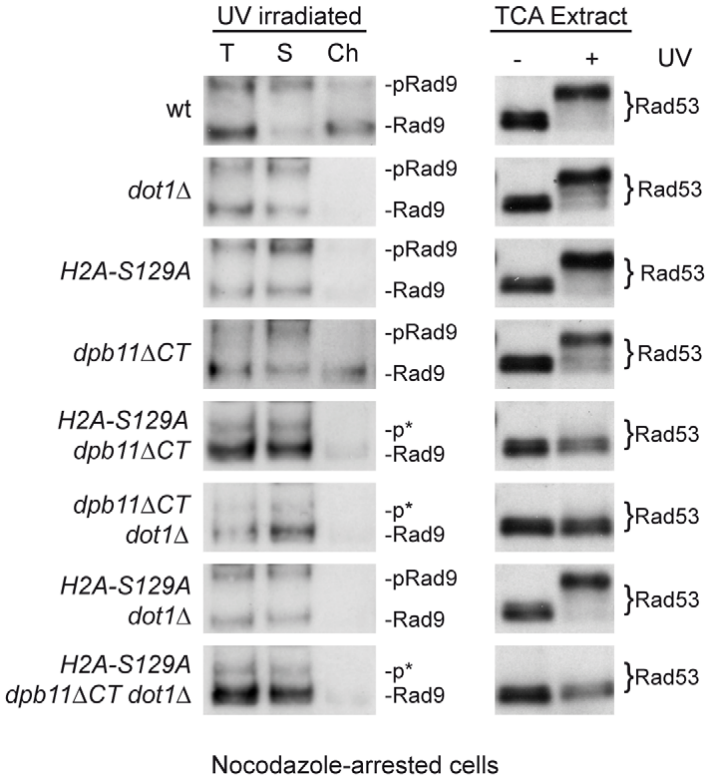
The Dpb11-dependent pathway in M phase modulates Rad53 activation in a chromatin-independent manner. wt (YMAG149/7B), *H2A-S129A* (YMAG168), *dpb11ΔCT* (YMAG145/20C), *dot1Δ* (YMAG150/4A), *H2A-S129A dpb11ΔCT* (YMAG155), *H2A-S129A dot1Δ* (YMAG170), *dpb11ΔCT dot1Δ* (YMAG148) and *H2A-S129A dpb11ΔCT dot1Δ* (YMAG157) strains were arrested in M with nocodazole and UV irradiated (75 J/m^2^). After 10 min, samples were collected and analyzed in their total (T), soluble (S) and chromatin-enriched (Ch) fractions; blots were probed with anti-Rad9 antibodies (left panel). Protein extracts were also prepared from mock and UV treated samples and analyzed by SDS-PAGE and Western blotting with anti-Rad53 antibodies to monitor checkpoint activation (right panel). The positions of Rad9 and its hyper-phosphorylated isoform (pRad9) are indicated. p* marks partially phosphorylated Rad9 species.

As shown in Figure 7, following DNA damage, the Dpb11 C-terminal region carrying the BRCT domain does not appear to be required for Rad9 binding to chromatin, as *dpb11DCT* cells behave as wild type. However, as expected, Rad9 chromatin recruitment is defective in *dot1Δ* and *H2A-S129A* mutant cells, as binding of Rad9 is dependent upon H3-K79me and γH2A, via its Tudor and BRCT domains respectively [22], [34], [60]. Checkpoint activation, as determined by Rad53 phosphorylation, was abolished in any double or triple mutant combinations carrying the *dpb11ΔCT* mutation (Figure 7). Intriguingly, even when detectable Rad9 binding to chromatin is abrogated (as in the single *dot1Δ* and *H2A-S129A* or in the double *dot1Δ H2A-S129A* mutant strains) Rad53 can be fully phosphorylated. Similar genetic dependencies were found when the various single, double and triple mutant strains were tested for checkpoint activation in response to zeocin treatment, which is known to cause DSBs (Figure S3 and data not shown).

### Dpb11 is responsible for checkpoint activation in M phase cells when the Rad9 BRCT domains are replaced with a heterologous dimerization domain

**Figure 8:**
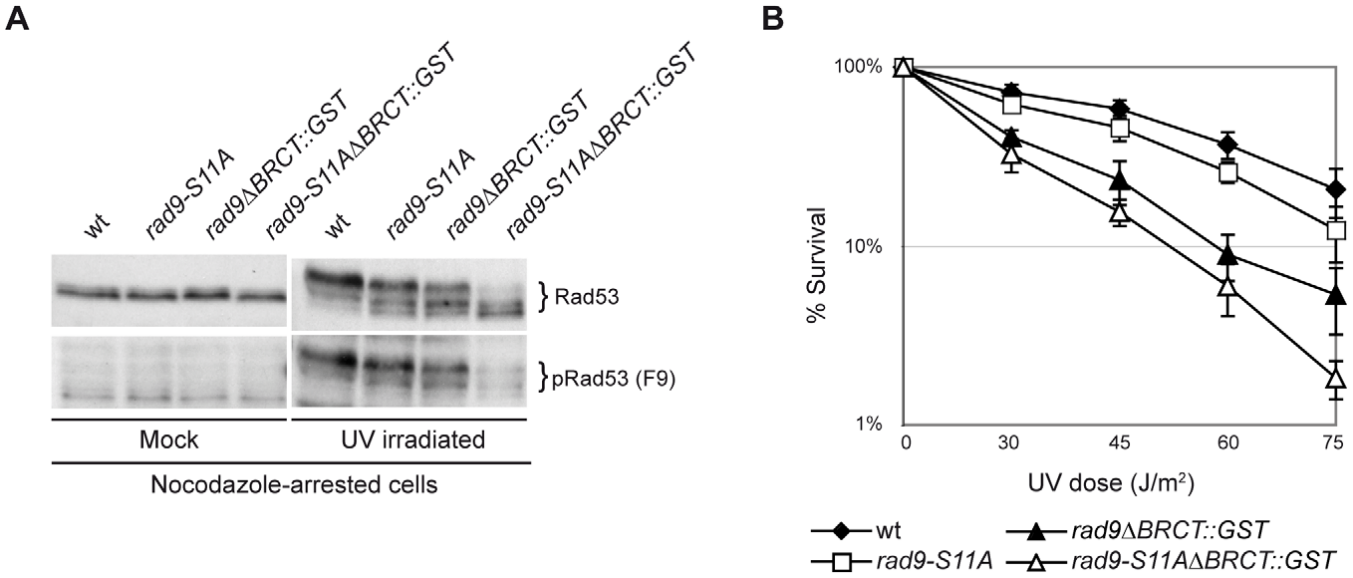
Partial checkpoint activation after forced Rad9 dimerization in M phase acts through the Dpb11-dependent checkpoint pathway. (A) wt (K699), *rad9-S11A* (YMAG162), *rad9ΔBRCT::GST* (YMAG74) and *rad9-S11AΔBRCT::GST* (YFL1177) strains were arrested with nocodazole and mock or UV irradiated (75 J/m^2^). After 10 min, samples were collected and protein extracts were separated by SDS-PAGE. Blots were analyzed either with anti-Rad53 antibodies or with the F9 Mab to monitor checkpoint activation. (B) UV survival assay. The same strains as in A were cultured overnight and then diluted and plated on YPD plates, which were irradiated with the indicated UV doses. Cell survival was assayed as described in the legend to Figure 3.

We have determined (Figure 3A) that in nocodazole-arrested cells defective checkpoint activation due to the absence of the Rad9 BRCT domain can be partially rescued by adding the GST dimerization domain. Moreover, we demonstrated that the M phase-specific DNA damage checkpoint contains a pathway based on Rad9-Dpb11 interactions and modulated via phosphorylation of the Ser11 residue of Rad9 by CDK1 (Figure 4, Figure 5, and Figure 6). As a consequence, we tested whether, in nocodazole-arrested cells, checkpoint activation supported by the heterologous dimerization motif in the *rad9ΔBRCT::GST* mutant strain was dependent upon Dpb11. To address this question, we introduced the *S11A* point mutation in the *rad9ΔBRCT::GST* strain (*rad9-S11AΔBRCT::GST)*. Whilst either single mutant strain was only partially defective in Rad53 phosphorylation, in *rad9-S11AΔBRCT::GST* cells, checkpoint activation was severely impaired (Figure 8A). This result indicates that in *rad9ΔBRCT::GST* cells residual checkpoint activation depends upon an active Dpb11 branch acting through a potential CDK1 site (S11) in the amino terminus of Rad9. As expected, *rad9-S11ADBRCT::GST* cells, in which the sole Rad9 expressed contains both the point mutation and the domain swap, are more sensitive to UV irradiation than either single mutant (Figure 8B).

**Figure 9:**
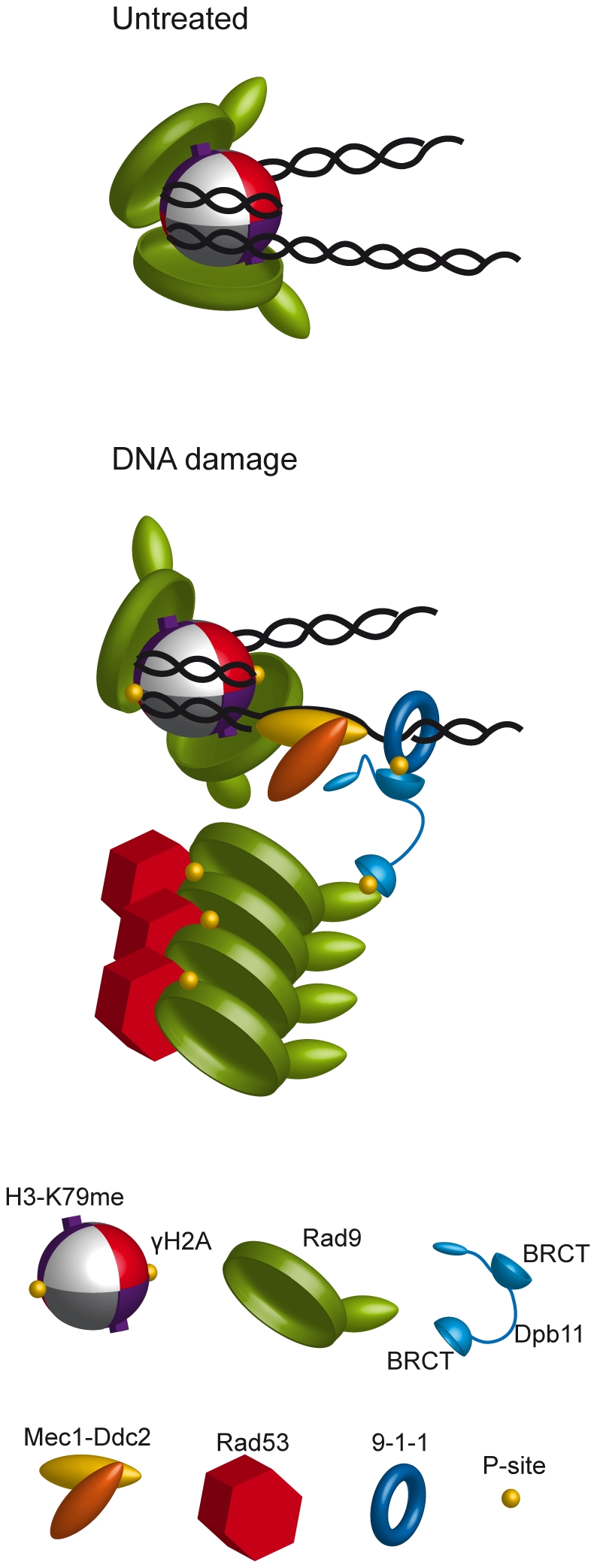
Possible model of the dynamics of Rad9 chromatin binding and its interaction with Dpb11 to modulate checkpoint activation in M phase. Under untreated conditions, Rad9 is chromatin bound through the interaction of its Tudor domain with H3-K79me and its BRCT-mediated dimerization. After DNA damage, activated Rad9 may change its conformation, interacting also with γ-H2A. In M-phase an alternative means of Rad9 recruitment near DNA lesions involves its interaction with Dpb11. This factor is brought near the Mec1-Ddc2 complex via its interaction with the 9-1-1 clamp, and it binds the phosphorylated N-terminal portion of Rad9 leading to full checkpoint activation.

In conclusion, our data are consistent with the hypothesis that Rad9 plays two independent roles in checkpoint activation: the first mediated by its dimerization and binding to modified histones, the second, which involves its phosphorylation by CDK1 and interaction with Dpb11 (Figure 9).

## Discussion

*RAD9* was the first DNA damage checkpoint gene identified in yeast [18]; however, the precise molecular details regarding the role of the corresponding gene product, its function and regulation remain far from being fully understood. In budding yeast, Rad9 seems to act as an adaptor protein in the signal transduction checkpoint cascade, mediating the transmission of the signal from the apical PIKKs to the main primary transducer kinase, Rad53 [27], [61]. Rad9 phosphorylation, mediated by Mec1, is an early event in the signal transduction cascade and this modification in G1 is mainly influenced by histone H3 methylation [22], [33], [60], [62]. In M phase, Rad9 phosphorylation also requires Dpb11, whose role as an alternative scaffold for Rad9 activation has been unveiled only recently [50]. The dynamics of Rad9 recruitment at various cell cycle stages and the genetic dependencies controlling Rad9 interaction with DNA/chromatin and other proteins are largely unknown.

Here, we show that a significant proportion of Rad9 is already chromatin-bound in unperturbed conditions throughout the cell cycle, confirming previous suggestions [22] and supporting our earlier model [14]. According to the current view, Rad9-chromatin association is controlled by interaction between its Tudor domain and H3-K79me. Constitutive, dynamic recruitment of Rad9 to chromatin may facilitate the efficiency and speed of the Rad9-dependent response to genotoxins. After DNA damage, Rad9 binding to chromatin is further strengthened through its BRCT domain, which is required to productively interact with γH2A [22], [23]. In this study we found that the BRCT domain of Rad9, in addition to promoting interaction with γH2A, has a more general function in modulating Rad9 recruitment. In fact, the *rad9-F1104L* and *rad9-W1280L* mutations, affecting the folding of the whole BRCT domain [21], alter binding to chromatin also in the absence of any genotoxic treatment. The observation that *rad9-K1088M* cells are defective in Rad9 chromatin recruitment only after γ-irradiation may be explained if such mutation only prevents Rad9-γH2A interaction [22].

In G1 cells, Rad9 binding to chromatin can be achieved by substituting the BRCT repeats with a heterologous dimerization domain; such recruitment requires the activity of Dot1 histone methyl-transferase, indicating that BRCT-mediated dimerization may be a pre-requisite for constitutive interaction between the Rad9 Tudor domain and H3-K79me. Given the symmetrical structure of the histone octamer within the nucleosome core, dimerization might facilitate the correct orientation and positioning of two Rad9 molecules on the nucleosome, allowing productive interactions with modified histones (Figure 9). Such hypothesis is supported by structural modeling of a dimeric *S. pombe* Crb2 complex on a single nucleosome, where all the interactions with H4-K20me and γH2A are satisfied without changing the conformation of the histone core [31].

It is worth noting that dimerization forced by replacement of the Rad9 BRCT domains with the heterologous GST tag only restores Rad9 binding to chromatin in G1-, and not in M-arrested cells. In fact, in cells arrested with nocodazole, we observed that GST-induced dimerization can rescue Rad9 hyper-phosphorylation and DNA damage checkpoint activation, but not its stable recruitment to chromatin. It is possible that in mitosis cell cycle-dependent phosphorylation of Rad9 may interfere with the chromatin association of this artificial Rad9 dimer. Alternatively, in nocodazole-arrested cells the Rad9 BRCT motifs may play additional roles in modulating Rad9-chromatin interactions.

Several findings indicate that the cellular response to DNA damage, including the repair mechanisms themselves, are regulated differently in distinct cell cycle stages. Multiple layers of cell cycle regulation may modulate the recruitment of critical checkpoint and repair factors to damaged DNA, as well as facilitate their reciprocal cross-talk [63]–[67]. We have previously shown that Dpb11 is essential for full DNA damage checkpoint activation in M-arrested cells [50]. Dpb11 is held in proximity to damaged DNA through its interaction with phosphorylated 9-1-1 complex, leading to Mec1-dependent Rad9 phosphorylation. Taking advantage of the *cdc28-as1* mutation, which allows conditional turn off of CDK1 kinase activity, we have demonstrated that CDK1, targeting Rad9, is required for the function of the Dpb11-dependent branch of the checkpoint response. Indeed, yeast cells carrying a truncated Rad9 version lacking 9 putative Cdc28 phosphorylation sites in the N-terminal region, are checkpoint-defective in M phase, in the absence of the histone-dependent branch. The Ser11 residue in the Rad9 N-terminal region is the most relevant Cdc28 target site, since a *rad9-S11A* mutation recapitulates the phenotypes observed in *rad9ΔNT* cells.

By two-hybrid analysis we showed that Rad9 and Dpb11 specifically interact in M-phase arrested cells, even in the absence of DNA damage, and this interaction is stimulated by CDK1-dependent Rad9 phosphorylation. Co-immunoprecipitation experiments confirmed that Rad9-Dpb11 interaction requires phosphorylation of Rad9-S11 and revealed that it depends upon genotoxic treatment, although we cannot exclude a weak/transient interaction in untreated conditions. This finding can be explained if activation of Mec1 by DNA damage facilitates or controls this interaction, e.g phosphorylating Dpb11 [50], exposing phospho-S11 or stimulating Rad9-S11 modification by CDK1. The overexpression conditions typical of the two hybrid system can easily explain why a weak interaction can be detected also in the absence of DNA damage. Interestingly, the functional interactions between Dpb11 and Rad9 in budding yeast are reminiscent of similar findings in the distantly related *S. pombe*, where histone-independent checkpoint activation is also modulated by CDK1 [37].

The Dpb11-dependent pathway does not require the histone modifications modulating Rad9 recruitment to chromatin. We found that a truncated C-terminal version of Dpb11 does not affect Rad9 recruitment to chromatin, which is instead abolished when the histone-dependent pathway is defective. Surprisingly, in a *dot1Δ H2A-S129A* double mutant strain checkpoint activation in M phase is virtually undistinguishable from that found in wild type cells, although Rad9 is not stably bound to chromatin. Only when the *dpb11ΔCT* mutation is combined with the *dot1Δ* or *H2A-S129A* mutation the checkpoint response is turned off. The working model presented in Figure 9, suggests that Dpb11 may act in M-phase as an alternative means of Rad9 recruitment. Dpb11 is located close to sites of DNA damage through its interaction with the Mec1-phosphorylated 9-1-1 complex; DNA damage leads to Mec1-dependent phosphorylation of Dpb11 [50], which interacts with S11-phosphorylated Rad9 (Figure 9). This Dpb11-dependent localization of Rad9 to sites of DNA damage allows rapid Rad9 hyper-phosphorylation by PIKKs, as suggested by the observation that the interaction between Rad9 and Dpb11 is induced by genotoxic agents and hyper-phosphorylated Rad9 is enriched in the Dpb11-bound population. Subsequently, Rad53 recruitment via its FHA domains leads to full activation of the checkpoint response. Unlike Rad9 bound via histone marks, Rad9 complexed with Dpb11 does not appear to be tightly linked to chromatin, explaining why the Dpb11-dependent branch for checkpoint activation seems to act in a chromatin-independent manner. However, we cannot rule out the possibility that the Rad9-Dpb11 complex can transiently or weakly bind to chromatin.

The model suggested here is in agreement with similar findings in the distantly related *S.pombe* fission yeast [37] as well as with recent *in vitro* data describing Dpb11 role in checkpoint activation [68], suggesting that the proposed mechanism can be extended to other eukaryotic organisms.

## Materials and Methods

### Strains and plasmids

All of the strains used in this work are derivatives of W303 [*MAT*a *ade2*-*1 trp1*-*1 can1*-*100 leu2*-*3*,*12 his3*-*11*,*15 ura3 rad5-535*]; only strains YFP91 and DLY2236 (provided by D. Lydall), are *RAD5^+^*. All the strains used in this study are listed in Table S1 and further information regarding strains and plasmids is available upon request.

Plasmids pMAG11.1 and pFP15 are, respectively, the Rad9 prey and Dpb11 bait plasmids used for the yeast two-hybrid analysis. They were obtained by amplifying the relevant coding sequences from genomic DNA and by ligating the resulting fragments into pJG4-5 and pEG202 [69], respectively.

The plasmid pMAG9, which encodes the Rad9ΔNT prey, was obtained cloning the *rad9ΔNT* sequence, amplified from the yeast strain DLY2236, into pJG4-5.

Gene deletions were obtained by PCR-mediated gene replacement [70].

The YNOV15 (*rad9-F1140L*) and YNOV31 (*rad9-W1280L*) strains were obtained from YFL871. The *kanMX4* and *KlURA3* CORE cassettes, amplified from pCORE [71], were integrated in a K699 strain at position 1941 of the *RAD9* gene. Subsequently, the CORE cassette was replaced with the C-terminus of the *rad9-F1104L* or *rad9-W1280L* alleles, amplified respectively from pFL75.5 or pFL69.1, thus restoring the full-length *RAD9* open reading frame bearing the intended mutation. *RAD9* site-specific mutations on plasmids pFL75.5 and pFL69.1 were obtained by PCR with mutagenic oligonucleotides on the pFL36.1 plasmid [50]. Recombination events were selected on 5-fluoroorotic acid plates, and the strains were verified by sequencing.

The *rad9ΔBRCT::13MYC* and the *rad9ΔBRCT::GST* mutant alleles were obtained by introducing the 13-MYC or GST tags at the 984 aa, using the one-step PCR method [70], thus eliminating the whole Rad9 BRCT domain.

The *cdc28-as1* mutant allele was obtained by ClaI-directed integration of plasmid pVF6 [72] at the *CDC28* locus into the desired background. Plasmid pop-out events were selected on 5-fluoroorotic acid plates, and the presence of the *cdc28-as1* mutation was verified by assessing sensitivity to 1NMPP1 on plate.

Strains encoding the *rad9-S11A* mutant allele were obtained by MscI-directed integration of pRS306-NTRAD9^cdk1^ into the desired background. The transversion TCT-GCT causing the *rad9-S11A* mutation and the reversion GCT to ACT generating the *rad9-S11T* allele were produced by site- directed mutagenesis (Stratagene) of pGEMTeasyRAD9, containing a 2547 bp fragment from position -445 to position +2102 within the *RAD9* ORF. The 1.8 Kb BamHI-MscI fragment from the pGEMTeasyRAD9 vector was swapped with the equivalent fragment from an existing 6.3 Kb pRS306-NTRAD9 integrative vector, containing a BamHI-SpeI *RAD9* fragment from position −445 to position 1478 within the *RAD9* ORF and the presence of the mutation verified by sequencing. Plasmid pop-out events were selected on 5-fluoroorotic acid plates, and the *rad9-S11A* mutation was confirmed by PCR sequencing.

The *dpb11ΔCT* mutant allele was obtained by introducing a premature stop codon at the 583 aa and the *HPH* cassette after the codon with the one step PCR method previously described [73], thus mimicking the *dpb11-1* mutation [74].

Strain YFL921 was obtained by using the one-step PCR strategy described in Longtine 1998, using pFA6-*FKBP2x-13MYC-KanMX6*, as template. This plasmid was generated by cloning in PacI-linearized pFA6-*13MYC-KanMX6* the *FKBP2x* sequence amplified from pC4M-FV2E (ARGENT Regulated Homodimerization kit, ARIAD Pharmaceutical).

The yeast two hybrid was performed using the B42/lexA system with strain EGY42 (*MATa his3 ura3 trp1 6lexAOP-LEU2*; *lex- AOP-lacZ* reporter on plasmid pBH18-34) as the host strain [69].

### Chromatin binding

To analyze chromatin binding of proteins, yeast extracts were prepared from G1- or M-arrested cells following published procedures [22].

### Cell cycle blocks and DNA damage treatments

Cells were grown in YPD medium at 28°C (25°C in the experiments with strains harboring the *dpb11ΔCT* mutation) to a concentration of 6×10^6^ cells/ml and arrested in G1 or M with α-factor (20 µg/ml) or nocodazole (20 µg/ml), respectively. 50 ml of cultures were centrifuged, resuspended in 500 µl of fresh YPD and plated on a Petri dish (14 cm diameter). Plates were quickly irradiated with a Stratalinker at 75 J/m^2^ and cells resuspended in 50 ml of YPD plus α-factor or nocodazole. A 25 ml sample was taken 10 min after the treatment and processed for protein extraction with trichloroacetic acid (TCA) [75]. For analysis of the double-strand breaks (DSBs) checkpoint response, cells arrested at the proper cell cycle phase were treated with 150 µg/ml of zeocin. Samples were taken 45 min after treatment and processed for protein extraction.

### FKBP dimerization

To analyze FKBP-driven (FK506 binding protein) dimerization, overnight cell cultures were diluted at a concentration of 1×10^6^ cells/ml and treated for 6 h with 1 µM AP20187 (ARGENT Regulated Homodimerization kit, ARIAD Pharmaceutical). UV sensitivity assays or chromatin binding analysis were performed as described elsewhere in this section.

### Inactivation of the Cdc28 kinase activity

Exponentially growing cells in a *cdc28-as1* background were harvested at a concentration of 4×10^6^ cells/ml and blocked in M phase as described above. To selectively inhibit Cdc28 activity [56], the ATP analogue 1NMPP1 was then added to a concentration of 5 µM to half of the cultures; after 2 h of incubation at 28°C, cells were either mock- or UV-irradiated and protein extracts were prepared.

### SDS-PAGE and western blotting

TCA protein extracts or chromatin binding samples were separated by sodium dodecyl sulfate-polyacrylamide gel electrophoresis (SDS-PAGE) in 10% acrylamide gels. For the analysis of Rad9 phosphorylation, NuPAGE Tris-acetate 3% to 8% gels were used following the manufacturer's instructions. Western blotting was performed with anti-Rad9 (D. Stern), anti-Rad53 (C. Santocanale), with anti-phosphorylated Rad53 F9 Mab antibodies [76] anti-ORC2 (Abcam) and anti-tubulin (ML. Carbone), using standard techniques.

### UV–sensitivity assay

To assess cell survival after UV irradiation, serials dilutions of overnight cultures were spotted onto YPD plates, which were either irradiated with different UV doses or mock-treated. For survival curves, yeast strains were cultured overnight to exponentially growing phase. Cells were diluted and approximately 500 cells/plate were plated, and then either irradiated with various UV doses or mock-treated. After 3 days, the total number of colonies formed on each plate was counted.

### Yeast two-hybrid analysis

Protein interaction between Rad9 and Dpb11 in the G1 and M phase of the cell cycle was assessed by measuring β-galactosidase activity with ortho-Nitrophenyl-β-galactoside (ONPG) assay. Briefly, cells expressing Rad9 bait and/or Dpb11 prey were cultured overnight in yeast synthetic media (-Ura, -His, -Trp) with 2% (w/v) raffinose to a concentration of 5×10^6^ cells/ml. Cultures were centrifuged and cells resuspended in YP plus raffinose and arrested in G1 or M phases, as described above. Galactose to a 2% w/v final concentration was added to the medium to induce prey expression. A 15 ml sample was taken after 1 h of galactose induction, centrifuged and resuspended in 250 µl of breaking buffer (100 mM Tris HCl at pH 8.0, Glycerol 10%; DTT 1 mM, 1 tablet of complete Roche antiproteolytic cocktail. Cells were lysed by using a FastPrep cell disruptor; the optical density (OD) of protein extract at 600 nm was determined using the Bio-Rad protein assay reagent. 1 ml of Z buffer (60 mM Na_2_HPO_4_, 40 mM NaH_2_PO_4_, 10 mM KCl, 1 mM MgSO_4_, and 50 mM β-mercaptoethanol at pH 7.0) plus ONPG 4 mg/ml was aliquoted in a small glass tube for each sample. 20 µl of protein extract was added to each tube and incubated at 37°C until a yellow color developed. The reaction was stopped by adding 400 ml of 1 M NaCO_3_ and the OD at 420 nm of each sample was measured. β -Galactosidase activity was calculated by using the formula units  = 10^3^ OD_420_/(OD_600_ x reaction time in min).

### Rad9-Dpb11-MYC immunoprecipitation

1.5 l cultures of strains YFP38 and YMAG281 expressing, respectively, the tagged Dpb11-MYC fusion protein under the control of the endogenous *DPB11* promoter in a wild-type or *rad9S11A* mutant background were grown in YPD medium at a cell density of 1×10^7^ cells/ml. Cells were then arrested in M phase by addition of 10 µg/ml of nocodazole and were either mock treated or treated with 150 µg/ml of zeocin for 30 min. Cells were washed twice with pre-cooled ddH_2_O and once in 2× lysis buffer (300 mM KCl, 100 mM Hepes (pH 7.5), 20% glycerol, 8 mM β-mercaptethanol, 2 mM EDTA, 0.1% Tween20, 0.01% NP-40). Resuspended cells were frozen as droplets in liquid nitrogen. Aliquots of frozen cells were manually ground in a mortar in liquid nitrogen. One volume of 2× lysis buffer, containing a protein inhibitor cocktail (2.8 µM leupeptin, 8 µM pepstatin A, 4 mM PMSF, 50 mM benzamidine, 25 µM antipain, 4 µM chymostatin in ethanol) and phosphatase inhibitors (2 mM sodium fluoride, 1.2 mM β-glycerophosphate, 0.04 mM sodium vanadate, 2 mM EGTA, 10 mM sodium pyrophosphate), was added. Cell extract was clarified by a low speed centrifugation followed by additional centrifugation for 1 h at 42.000 rpm in a Beckman Sw55Ti rotor. The clarified crude extract (Ext) was adjusted to 10 mg/ml in the various immunoprecipitation experiments. 1 ml of Ext was pre-cleared by incubation with 40 µl of 50% (v/v beads/1× lysis buffer) Protein G slurry for 1 hour at 4°C on a rotating wheel. Pre-cleared supernatants were incubated with either 20 µg of the anti-myc Mab 9E11 or 20 µg of unspecific mouse IgG. Samples were incubated for 2 h at 4°C on a rotating wheel and centrifuged at 14.000 rpm for 15 min at 4°C. 40 µl of 50% protein G slurry were added to the supernatants, incubated on a rotating wheel for 2 h at 4°C and recovered by centrifugation. Immunoprecipitated Dpb11-MYC samples were washed four times with 1 ml of lysis buffer containing protease and phosphatase inhibitors. Beads were finally resuspended in 40 µl of 3× Laemmli buffer (IP), boiled for 5 min and released proteins separated on 6.5% (80/1 acrylamide/bisacrylamide) SDS-PAGE gels. After blotting, Rad9 was visualized with the NLO5 Rad9 polyclonal antibody [13] or the 9E11 Mab (Abcam).

## Supporting Information

Figure S1 (A) wt (K699) cells were arrested in G1 with α-factor and either mock or UV irradiated (75 J/m^2^). 10 min after irradiation, samples were collected and analyzed in their total (T), soluble (S) and chromatin-enriched (Ch) fractions. Blots were probed with anti Rad9 polyclonal antibodies. After UV irradiation the hyper-phosphorylated Rad9 isoform migrates and it is detected on Western blots probed with anti-Rad9 antibodies near to an aspecific protein species (mostly present in the supernatant fraction) [50]. Such band was omitted in the Western blots shown in Figure 1, Figure 2, and Figure 7 for clarity. The positions of Rad9 and its hyper-phosphorylated isoform (pRad9) are indicated; * marks the background protein species unrelated to Rad9. (B) The Western blots in which the presence of Rad9 was analyzed in the total (T), soluble (S) and chromatin-enriched (Ch) fractions were controlled for proper fractionation of control proteins, known to remain in the soluble fraction (Tubulin) or to bind to chromatin (Orc2). The blots in S1 Panel B show the results obtained with the same protein samples analyzed in Figure 1A. (C) Quantitative analysis of the percentage of hyper-phosphorylated and hypo-phosphorylated Rad9 isoforms in the total (T), soluble (S) and chromatin-enriched (Ch) fractions in α-factor and nocodazole arrested wild-type cells. Quantification was obtained with a Versadoc (Biorad) after incubation with fluorescent secondary antibodies, and error bars were obtained from 4 independent experiments. The percentages of hyper- and hypo- phosphorylated isoforms were calculated respectively to the total amount of Rad9.
(1.16 MB TIF)

Figure S2 (A) The histograms show the M/G1 ratio increase in β-galactosidase activity, when the interaction between Dpb11/Rad9 or the positive controls p53 and SV40-TAg was measured by two-hybrid analysis in nocodazole (M) or α-factor (G1) arrested cells. Error bars were obtained from three independent two-hybrid experiments. (B) Amino acid sequence of the Rad9 ORF; the basic CDK1 (S/T-P) and PIKK (S/T-Q) consensus phosphorylation sites are shown in black or gray, respectively. (C) wt (K699) and *rad9-S11A* (YMAG162) strains were arrested in M with nocodazole and samples were collected to prepare protein extracts. Rad9 phosphorylation was analyzed by SDS-PAGE and Western blotting with anti-Rad9 antibodies.
(0.77 MB TIF)

Figure S3 wt (YMAG149/7B), *H2A-S129A* (YMAG168), *dpb11ΔCT* (YMAG145/20C), *H2A-S129A dpb11ΔCT* (YMAG155), *dot1Δ* (YMAG150/4A), *H2A-S129A dot1Δ* (YMAG170), *dpb11ΔCT dot1Δ* (YMAG148) and *H2A-S129A dpb11ΔCT dot1Δ* (YMAG157) strains were arrested in M with nocodazole and treated with zeocin (150 µg/ml). After 45 min, samples were collected and protein extracts were analyzed by SDS-PAGE and Western blotting with anti Rad53 antibodies to monitor checkpoint activation.
(0.76 MB TIF)

Table S1 Strains used in this study. All of the strains used in this work are derivatives of W303 [*MAT*a *ade2*-*1 trp1*-*1 can1*-*100 leu2*-*3*,*12 his3*-*11*,*15 ura3 rad5-535*]; only strains YFP91 and DLY2236 (provided by D. Lydall), are *RAD5^+^.*
(0.06 MB DOC)
