# Correction: Dynamics of Rad9 Chromatin Binding and Checkpoint Function Are Mediated by Its Dimerization and Are Cell Cycle–Regulated by CDK1 Activity

**DOI:** 10.1371/journal.pgen.1004535

**Published:** 2014-06-27

**Authors:** The PLOS Genetics Staff

Figure 5 is incorrect. It contains components that are duplicated and do not correspond to what is stated in the figure legend or in the text. Specifically, the left pRad53 panel (mock) in Figure 5B is a duplication of the left pRad53 panel (mock) from Figure 5A. The authors have provided a corrected version here. This corrected version corresponds to what is stated in the figure legend and in the text.

**Figure 5:**
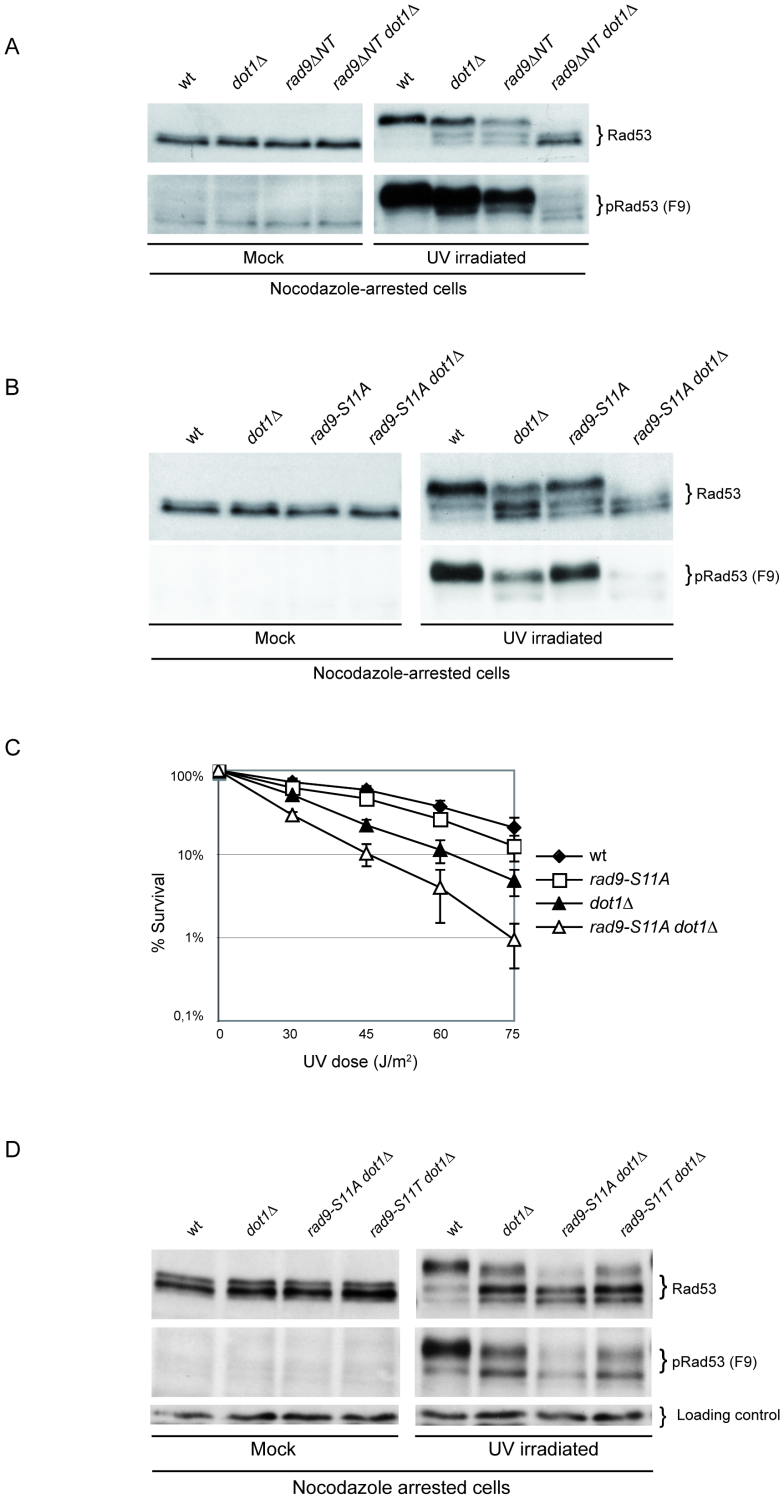
Phosphorylation of Rad9S11 by CDK1 is required for the establishment of an effective UV response in the absence of Dot1. (A) wt (K699), *dot1Δ* (YFL234), *rad9ΔNT* (DLY2236) and *rad9ΔNT dot1Δ* (YFP91) strains were arrested with nocodazole and either mock or UV irradiated (75 J/m^2^). After 10 min samples were collected and protein extracts were separated by SDS-PAGE. Blots were analyzed with anti-Rad53 or with the F9 Mab to monitor checkpoint activation. (B) wt (K699), *dot1Δ* (YFL234), *rad9-S11A* (YMAG162) and *rad9-S11A dot1Δ* (YMAG164) strains were arrested in M, irradiated and Rad53 was detected by Western blotting as describe in panel A. (C) The same strains analyzed in B were cultured overnight, diluted and plated on YPD plates, which were irradiated with the indicated UV doses. Cell survival was assayed as described in the legend of Figure 3. (D) wt (K699), *dot1Δ* (YFL234), *rad9-S11A dot1Δ*(YMAG164) and *rad9-S11T dot1Δ* (YNOV52) strains were arrested with nocodazole and either mock or UV irradiated (75 J/m^2^). After 10 min samples were collected and protein extracts were separated by SDS-PAGE..Blots were analyzed with anti-Rad53 or with the F9 Mab to monitor checkpoint activation.
