# Supplementary figures and images for: Dynamics of Rad9 Chromatin Binding and Checkpoint Function Are Mediated by Its Dimerization and Are Cell Cycle–Regulated by CDK1 Activity
Source: PLoS Genet. 2010 Aug 5;6(8):e1001047. doi: 10.1371/journal.pgen.1001047 (PMC2916856; doi:10.1371/journal.pgen.1001047)

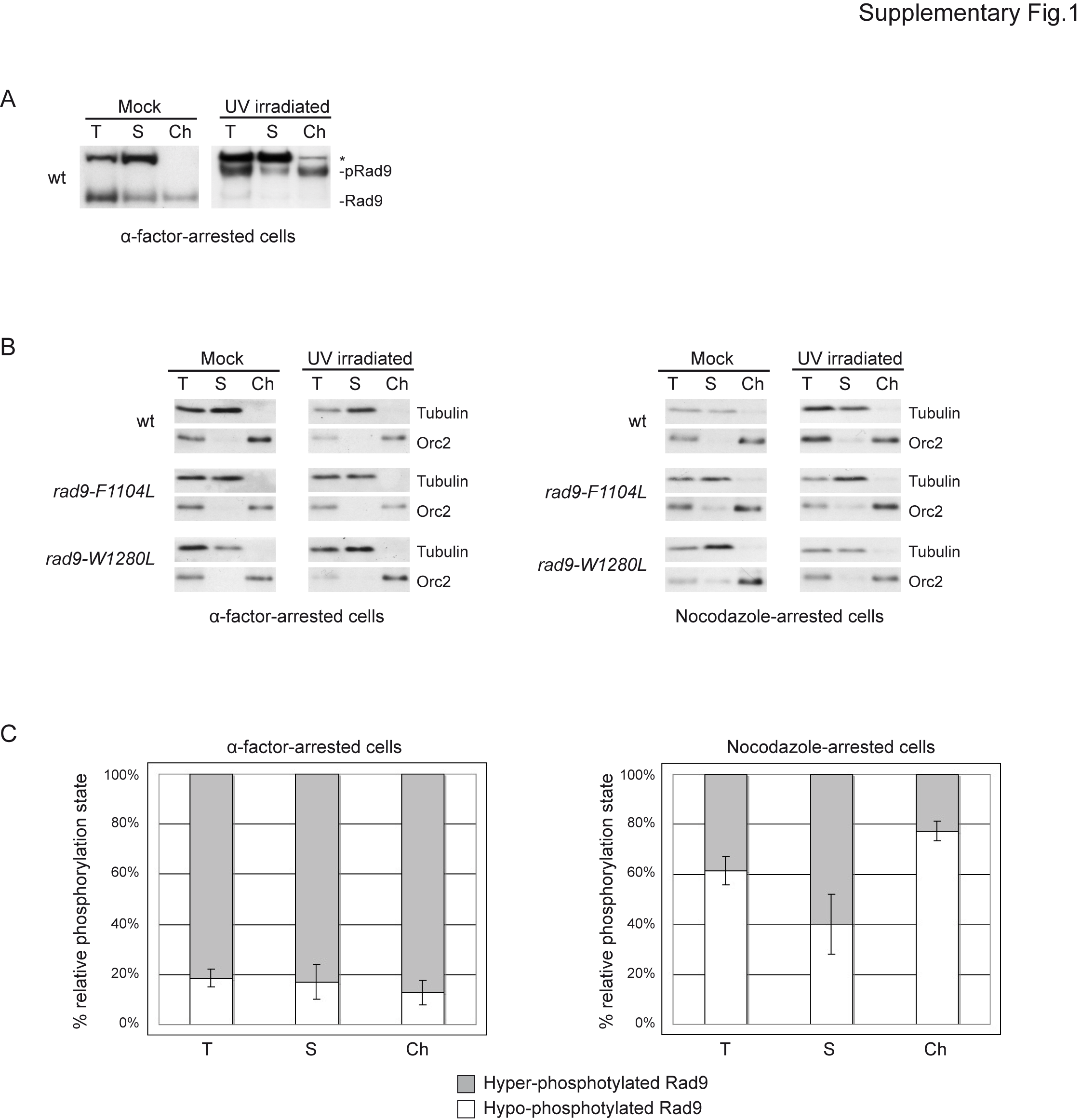

Supplement: Figure S1 — (A) wt (K699) cells were arrested in G1 with α-factor and either mock or UV irradiated (75 J/m2). 10 min after irradiation, samples were collected and analyzed in their total (T), soluble (S) and chromatin-enriched (Ch) fractions. Blots were probed with anti Rad9 polyclonal antibodies. After UV irradiation the hyper-phosphorylated Rad9 isoform migrates and it is detected on Western blots probed with anti-Rad9 antibodies near to an aspecific protein species (mostly present in the supernatant fraction) [50]. Such band was omitted in the Western blots shown in Figure 1, Figure 2, and Figure 7 for clarity. The positions of Rad9 and its hyper-phosphorylated isoform (pRad9) are indicated; * marks the background protein species unrelated to Rad9. (B) The Western blots in which the presence of Rad9 was analyzed in the total (T), soluble (S) and chromatin-enriched (Ch) fractions were controlled for proper fractionation of control proteins, known to remain in the soluble fraction (Tubulin) or to bind to chromatin (Orc2). The blots in S1 Panel B show the results obtained with the same protein samples analyzed in Figure 1A. (C) Quantitative analysis of the percentage of hyper-phosphorylated and hypo-phosphorylated Rad9 isoforms in the total (T), soluble (S) and chromatin-enriched (Ch) fractions in α-factor and nocodazole arrested wild-type cells. Quantification was obtained with a Versadoc (Biorad) after incubation with fluorescent secondary antibodies, and error bars were obtained from 4 independent experiments. The percentages of hyper- and hypo- phosphorylated isoforms were calculated respectively to the total amount of Rad9. (1.16 MB TIF) [file pgen.1001047.s001.tif]

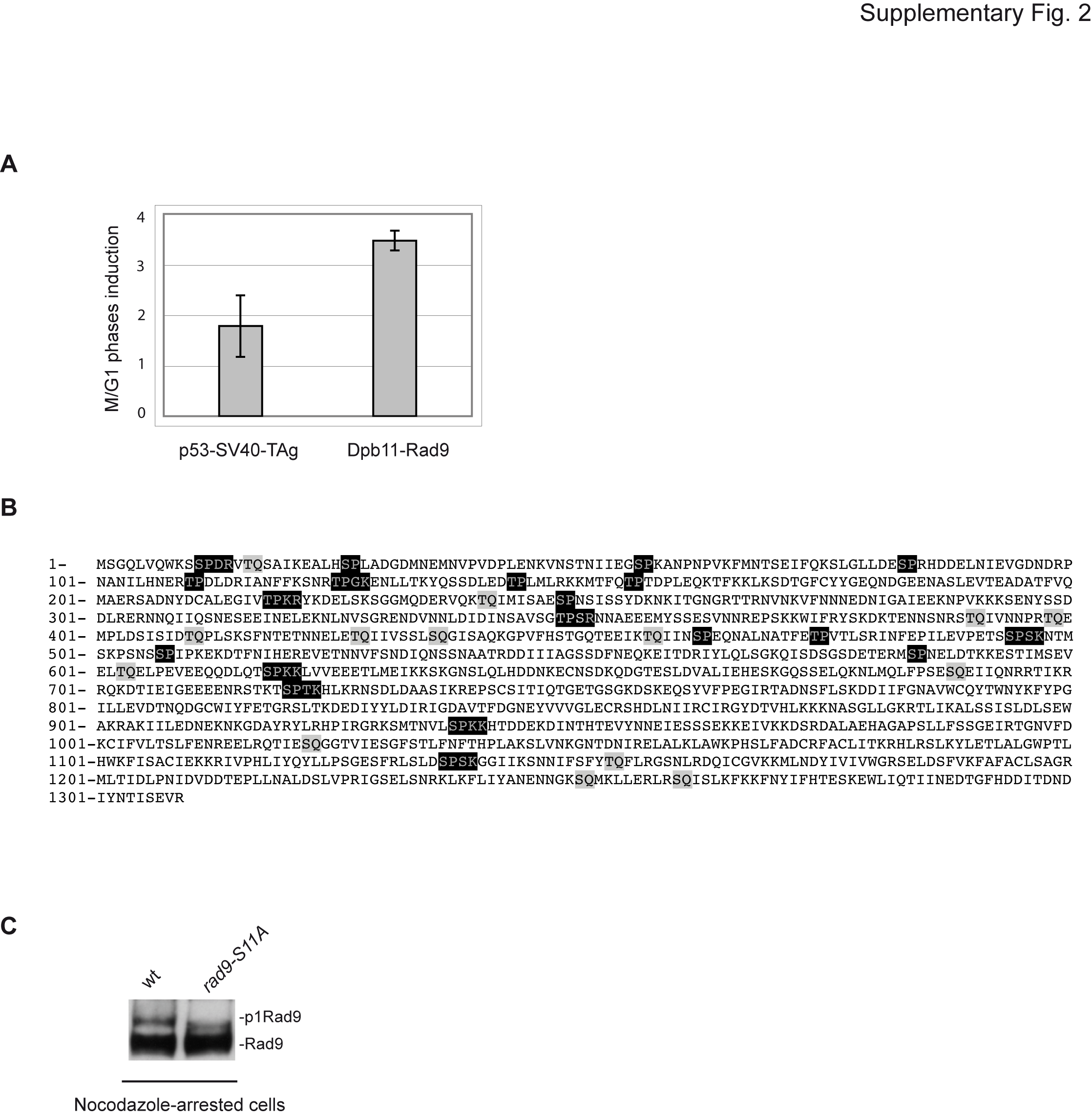

Supplement: Figure S2 — (A) The histograms show the M/G1 ratio increase in β-galactosidase activity, when the interaction between Dpb11/Rad9 or the positive controls p53 and SV40-TAg was measured by two-hybrid analysis in nocodazole (M) or α-factor (G1) arrested cells. Error bars were obtained from three independent two-hybrid experiments. (B) Amino acid sequence of the Rad9 ORF; the basic CDK1 (S/T-P) and PIKK (S/T-Q) consensus phosphorylation sites are shown in black or gray, respectively. (C) wt (K699) and rad9-S11A (YMAG162) strains were arrested in M with nocodazole and samples were collected to prepare protein extracts. Rad9 phosphorylation was analyzed by SDS-PAGE and Western blotting with anti-Rad9 antibodies. (0.77 MB TIF) [file pgen.1001047.s002.tif]

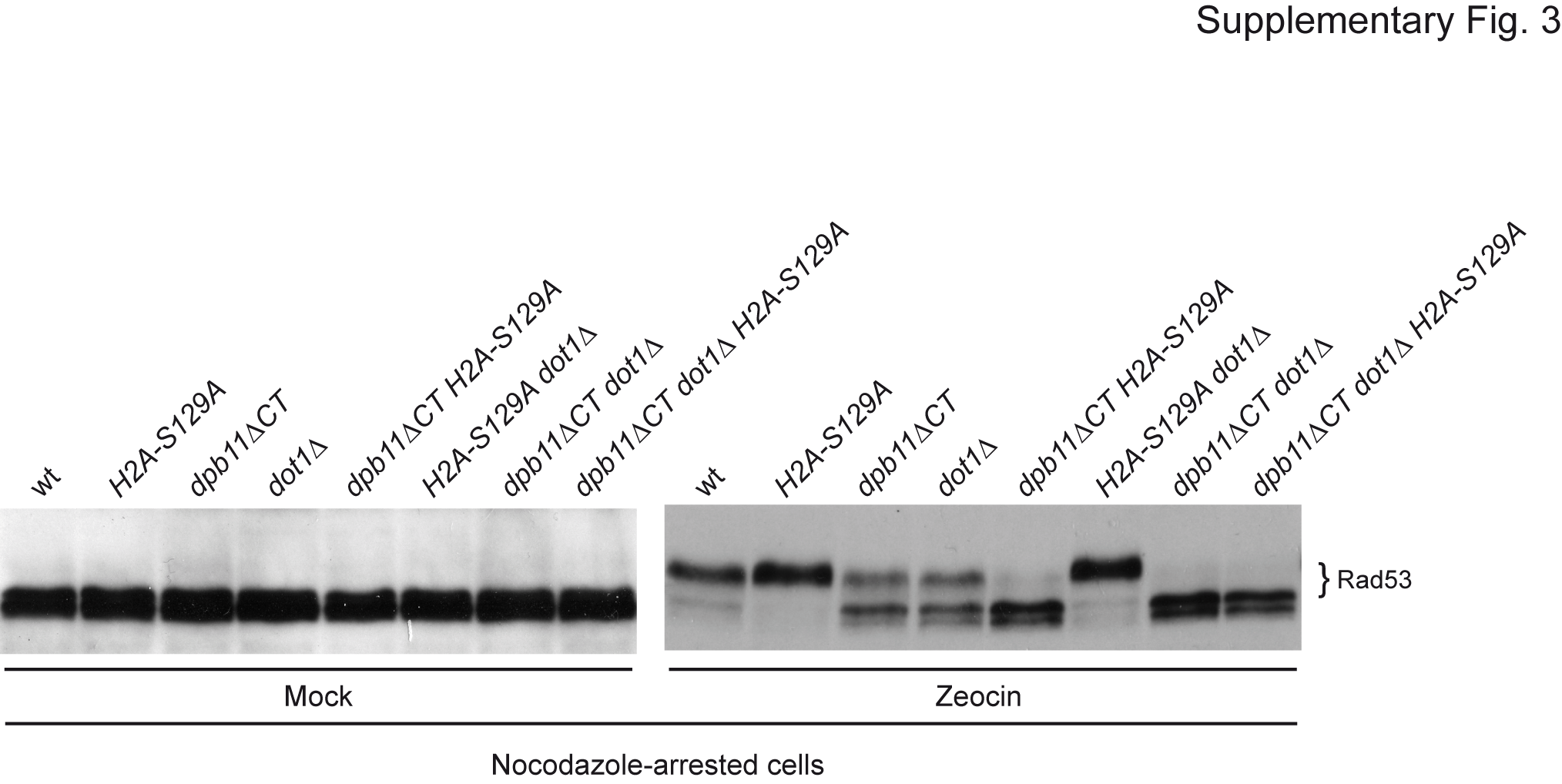

Supplement: Figure S3 — wt (YMAG149/7B), H2A-S129A (YMAG168), dpb11ΔCT (YMAG145/20C), H2A-S129A dpb11ΔCT (YMAG155), dot1Δ (YMAG150/4A), H2A-S129A dot1Δ (YMAG170), dpb11ΔCT dot1Δ (YMAG148) and H2A-S129A dpb11ΔCT dot1Δ (YMAG157) strains were arrested in M with nocodazole and treated with zeocin (150 µg/ml). After 45 min, samples were collected and protein extracts were analyzed by SDS-PAGE and Western blotting with anti Rad53 antibodies to monitor checkpoint activation. (0.76 MB TIF) [file pgen.1001047.s003.tif]
