# Supplementary material for: Dynamics of Rad9 Chromatin Binding and Checkpoint Function Are Mediated by Its Dimerization and Are Cell Cycle–Regulated by CDK1 Activity
Source: PLoS Genet. 2010 Aug 5;6(8):e1001047. doi: 10.1371/journal.pgen.1001047 (PMC2916856; doi:10.1371/journal.pgen.1001047)
Supplement: Table S1 — Strains used in this study. All of the strains used in this work are derivatives of W303 [MATa ade2-1 trp1-1 can1-100 leu2-3,12 his3-11,15 ura3 rad5-535]; only strains YFP91 and DLY2236 (provided by D. Lydall), are RAD5+. (0.06 MB DOC) [file pgen.1001047.s004.doc]

**Table 1.** Strains used in this study.

| **Strain** | Relevant genotype | **Source** |
| --- | --- | --- |
| K699 | *MAT*a *ade2-1 trp1-1 leu2-3,112 his3-11 ura3 can1-100 rad5-535* | K. Nasmyth |
| YFL871 | K699 *rad9aa1-646:kanMX4:URA3* | This work |
| YNOV15 | K699 *rad9-F1104L* | This work |
| YNOV31 | K699 *rad9-W1280L* | This work |
| YMAG88 | K699 *rad9::HIS3* | This work |
| YFL696/1b | K699 *rad9ΔBRCT::13MYC:TRP1* | This work |
| YMAG74 | K699 *rad9ΔBRCT::GST:kanMX6* | This work |
| YFL773/2c | K699 *dot1::kanMX6 rad9ΔBRCT::GST:kanMX6* | This work |
| YFL921 | K699 *rad9ΔBRCT::2xFKBP-13MYC:kanMX6* | This work |
| YMAG149/7B | K699 *hta1_htb1::LEU2 hta2_htb2::TRP1* (pSAB6) | (50) |
| YMAG145/20C | K699 *hta1_htb1::LEU2 hta2_htb2::TRP1 dpb11*Δ*CT::HPH* (pSAB6) | This work |
| YMAG150/4A | K699 *hta1_htb1::LEU2 hta2_htb2::TRP1 dot1::kanMX6* (pSAB6) | (50) |
| YMAG148 | K699 *hta1_htb1::LEU2 hta2_htb2::TRP1 dpb11*Δ*CT::HPH dot1::kanMX6* (pSAB6) | This work |
| EGY42 | *MAT*α *his3 ura3 trp1 leu2::6LexAop-LEU2* | R. Brent |
| JAU01 | K699 *cdc28-as1* | (56) |
| YNOV4 | K699 *cdc28-as1 dot1::kanMX6* | This work |
| YFL234 | K699 *dot1::kanMX6* | (33) |
| DLY2236 | K699 *rad9::LEU2 ura3::rad9-M232:URA3 RAD5+* | (58) |
| YFP91 | K699 *rad9::LEU2 ura3::rad9-M232-URA3 dot1::kanMX6 RAD5+* | This work |
| YMAG162 | K699 *rad9-S11A* | This work |
| YMAG164 | K699 *rad9-S11A dot1::kanMX6* | This work |
| YMAG168 | K699 *hta1_htb1::LEU2 hta2_htb2::TRP1* (pJD151) | (50) |
| YMAG170 | K699 *hta1_htb1::LEU2 hta2_htb2::TRP1 dot1::kanMX6* (pJD151) | (50) |
| YMAG155 | K699 *hta1_htb1::LEU2 hta2_htb2::TRP1 dpb11*Δ*CT::HPH* (pJD151) | This work |
| YMAG157 | K699 *hta1_htb1::LEU2 hta2_htb2::TRP1 dpb11*Δ*CT::HPH dot1::kanMX6* (pJD151) | This work |
| YFL1177 | K699 *rad9-S11AΔBRCT::GST:kanMX6* | This work |
